# Chlorogenic Acid Ameliorates Post‐Infectious Irritable Bowel Syndrome by Regulating Extracellular Vesicles of Gut Microbes

**DOI:** 10.1002/advs.202302798

**Published:** 2023-08-24

**Authors:** Cihua Zheng, Yuchun Zhong, Wenming Zhang, Zhuoya Wang, Haili Xiao, Wenjun Zhang, Jian Xie, Xiaogang Peng, Jun Luo, Wei Xu

**Affiliations:** ^1^ Department of General Surgery The Second Affiliated Hospital of Nanchang University 1 Minde Road Nanchang Jiangxi 330006 P. R. China; ^2^ Department of Rehabilitation Medicine The Second Affiliated Hospital of Nanchang University 1 Minde Road Nanchang Jiangxi 330006 P. R. China; ^3^ Jiangxi Province Key Laboratory of Molecular Medicine The Second Affiliated Hospital of Nanchang University Nanchang Jiangxi 330006 P. R. China

**Keywords:** PI‐IBS, *bacteroides acidifaciens*, chlorogenic acid, extracellular vesicles, glycine

## Abstract

Post‐infectious irritable bowel syndrome (PI‐IBS) occurs after acute infectious diarrhea, and dysbiosis can be involved in its pathogenesis. Here, the role of chlorogenic acid (CGA) is investigated, a natural compound with several pharmacological properties, in alleviating PI‐IBS in rats. It is elucidated that the gut microbiota plays a key role in PI‐IBS pathogenesis and that rectal administration of CGA alleviated PI‐IBS by modulating the gut microbiota and its metabolites. CGA supplementation significantly increased fecal *Bacteroides acidifaciens* abundance and glycine levels. Glycine structurally altered *B. acidifacien*s extracellular vesicles (EVs) and enriched functional proteins in the EVs; glycine‐induced EVs alleviated PI‐IBS by reducing inflammation and hypersensitivity of the intestinal viscera and maintaining mucosal barrier function. Moreover, *B. acidifacien*s EVs are enriched in the brain tissue. Thus, CGA mediates the mitigation of PI‐IBS through the gut microbiota and its metabolites. This study proposes a novel mechanism of signal exchange between the gut microenvironment and the host.

## Graphical abstract

The rectal administration of chlorogenic acid (CGA) reversed the decrease in *B. acidifaciens* and glycine in post‐infectious irritable bowel syndrome (PI‐IBS), and the combined administration of both can alleviate the symptoms of PI‐IBS. Glycine‐induced *B. acidifaciens* extracellular vesicles can reduce inflammation and hypersensitivity of the intestinal viscera, maintain mucosal barrier function, and be enriched in brain tissue to relieve PI‐IBS.

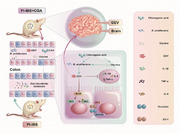

## 1 Introduction

Irritable bowel syndrome (IBS) is the most common gut–brain interaction disorder and is characterized by abdominal pain, bloating, irritability, and altered bowel habits.^[^ ^1^ ^]^ It manifests as colonic hypersensitivity, epithelial dysfunction, low‐grade mucosal inflammation, and disturbance of gut microbiota.^[^ ^2^ ^]^ The etiology of IBS is multifactorial, with gastrointestinal infections causing one‐third of IBS cases.^[^ ^3^ ^]^ After acute infectious diarrhea caused by bacteria, viruses, or parasites, 10%–30% of patients develop diarrhea‐predominant IBS symptoms, termed post‐infectious IBS (PI‐IBS).^[^ ^4^ ^]^ Although the detailed pathological mechanism of PI‐IBS is unclear, evidence suggests that gut microbiota disturbances are related to the development of PI‐IBS.^[^ ^5^ ^]^ The composition and abundance of the gut microbiota in patients with PI‐IBS are significantly different, and the levels of short‐chain fatty acids (SCFAs), which exhibit inhibitory activity against pathogenic bacteria, are also reduced due to inflammatory immune responses.^[^ ^6^ ^]^

The complex microbiota of the human gut affects host health, both directly and indirectly. The host provides a growth environment for the gut microbiota, which participates in maintaining host homeostasis by regulating intestinal development, nutrient digestion, and immune status, as well as affecting brain activity through the enteric nervous system.^[^ ^7^ ^]^ The drugs currently used for the clinical treatment of IBS mainly relieve symptoms and provide psychotherapy; however, effective treatments for curing IBS are not available.^[^ ^8^ ^]^ Chlorogenic acid (CGA) is a natural compound produced by plants during aerobic respiration, such as honeysuckle and *Eucommia*, and exhibits anti‐inflammatory, antioxidant, and neuroprotective properties.^[^ ^9^ ^]^ Wang et al. reported that CGA can improve anxiety, depression, and other posttraumatic stress disorder (PTSD)‐like symptoms.^[^ ^10^ ^]^ CGA modulates the gut microbiota and increases the abundance of SCFA‐producing bacteria.^[^ ^11^ ^]^ CGA treatment increases the relative abundance of *Bifidobacterium* spp. and the expression of the intestinal tight junction (TJ) proteins occludin and zonula occludens‐1 (ZO‐1), protects the intestinal barrier, and reduces the inflammatory response.^[^ ^12^ ^]^ Moreover, the bioavailability of CGA is largely dependent on its metabolism by the gut microbiota, and these microbial‐derived metabolites account for 57.4% of the total intake of CGA.^[^ ^13^ ^]^ Therefore, we hypothesized that CGA might alleviate the symptoms of PI‐IBS by interacting with the gut microbiota or exerting intestinal and neuroprotective effects.

*Bacteroides acidifaciens* is a gut‐dominant symbiont and belongs to the phylum *Bacteroidetes*. A soluble high‐fiber diet increases the relative abundance of *B. acidifaciens* in the gut microbiome and leads to enhanced tumor radiosensitivity.^[^ ^14^ ^]^ Furthermore, a recent study revealed that *B. acidifaciens* exerts a protective effect against ConA‐induced liver injury in a mouse model.^[^ ^15^ ^]^ Nagahara et al. revealed that *B. acidifaciens* could increase intestinal immunoglobulin A levels, protect against bowel pathogens and reduce inflammatory bowel diseases.^[^ ^16^ ^]^ Although many studies have revealed the protective effects of *B. acidifaciens* on organisms, understanding the interaction of intestinal microorganisms and their metabolites with *B. acidifaciens* requires further research. The exchange of biological signals between prokaryotic and eukaryotic cells is largely mediated by the secretion of extracellular vesicles (EVs).^[^ ^17^ ^]^ The production of EVs is ubiquitous in gram‐negative bacteria and some gram‐positive bacteria, and EVs (20–400 nm) derived from the gut microbiota are important for cellular communication, as they carry functional molecules that can modulate the function of the recipient cells.^[^ ^18^ ^]^ Yang et al. revealed that *Lactobacillus reuteri* EVs can improve lipopolysaccharide (LPS)‐induced intestinal injury and the inflammatory response, confirming the necessity of proteins and nucleic acids in EV‐mediated bacteria–host interactions.^[^ ^19^ ^]^ Xie et al. reported that *Akkermansia* (*Akk*) could communicate with host bone cells by secreting EVs, which penetrate the host intestinal barrier and are transported to bone tissue to mediate bone protection.^[^ ^18^ ^]^ This suggests that bacterial EVs may be the key mechanism underlying the gut microbiota‐regulated gut–brain interaction in diseases.

In this study, we investigated the role of gut microbiota in a PI‐IBS rat model and studied whether CGA exerts a relieving effect on PI‐IBS symptoms. We used 16S ribosomal RNA (rRNA) gene sequencing and untargeted metabolomics to screen candidate bacteria and metabolites that may be associated with the effects of CGA on PI‐IBS symptoms and validated the results by transplantation of candidate bacteria (*B. acidifaciens*) and metabolites (glycine). In vitro experiments revealed that bacterial EVs may be a potential link between *B. acidifaciens* and glycine. Finally, we determined whether the EVs produced by the gut microbiota, induced by specific metabolites, play a regulatory role in the development of inflammatory responses, gut visceral hypersensitivity, and mucosal barrier function in PI‐IBS rats. Furthermore, we performed a proteomic analysis of nonglycine‐induced (EV) or glycine‐induced (GEV) EVs to determine whether there were differences in the functional proteins of EVs and GEVs.

## 2 Results

### 2.1 Gut Microbiota Disturbances Observed in PI‐IBS Rats

**Figure 1:**
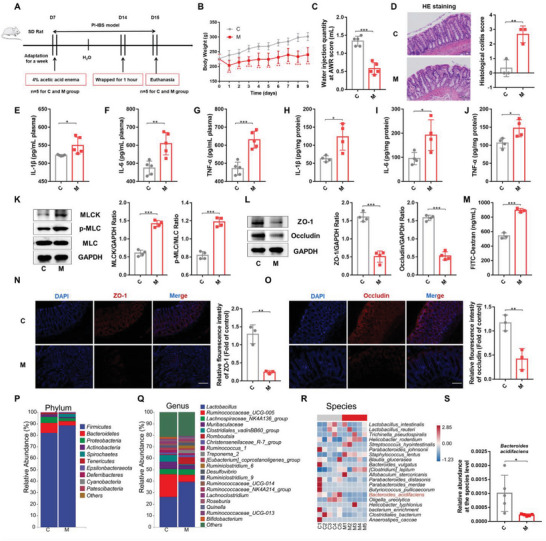
Rectal administration of AA induced PI‐IBS and caused gut microbiota disorder in rats. A) Experimental scheme of the PI‐IBS rat model used in this study (*n* = 5). B) Changes in body weight of PI‐IBS rats (*n* = 5). C) Water injection rate when the AWR score was 3 (*n* = 5). D) H&E staining and pathological score of colon sections (*n* = 3). E–J) Concentrations of the representative proinflammatory cytokines IL‐1β, IL‐6, and TNF‐α in rat serum samples of colon tissue (*n* = 4‐5). K) Expression of key proteins of the MLCK/p‐MLC signaling pathway in rat colon tissue samples (*n* = 4). L) The expression of TJ proteins (ZO‐1, Occludin) in rat colon tissue (*n* = 4). M) Measurement of gut permeability in rats (*n* = 3). N and O) Immunofluorescence staining of TJ protein (ZO‐1, Occludin) expression in the colonic section (*n* = 3). Q,R) Relative abundance of the identified fecal microbiota at the (P) phylum, (Q) genus, and (R) species levels as per 16S rRNA gene sequencing (*n* = 5). S) The relative abundance of *B. acidifaciens*. C, control group; M, PI‐IBS rat group. Scale bar, 100 µm. Data are presented as the mean ± SD. ns, *p* > 0.05; * *p* < 0.05; ** *p* < 0.01; *** *p* < 0.001. AA: Acetic acid; PI‐IBS: Post‐infectious irritable bowel syndrome; AWR: Abdominal withdrawal reflex; TJ: Tight junction; PCoA: Principal coordinate analysis.

To induce PI‐IBS in rats, we rectally administered a single dose of 4% acetic acid (AA) and, after 7 days, stimulated the rats with bondage stress (**Figure** **1**A). As shown in Figure 1B and C, compared to that in the control (C) group, rectal administration of AA significantly reduced body weight and increased visceral sensitivity. Histological analysis revealed that inflammatory cells infiltrated the colon, intestinal villi were shortened and thickened, and mucosa was damaged in response to 4% AA treatment (Figure 1D). To evaluate the systemic inflammatory response in PI‐IBS rats, the serum and colon tissue levels of proinflammatory cytokines were measured. Compared to that in the C group, the expression of interleukin (IL)−1β, IL‐6, and tumor necrosis factor (TNF)‐α in PI‐IBS rats (M group) was significantly increased at the protein level (Figure 1E–J). Furthermore, we detected the upregulation of myosin light chain kinase (MLCK) and phosphorylated myosin light chain (p‐MLC) and the downregulation of intestinal epithelial TJ proteins (ZO‐1, occludin) in AA‐stimulated rats by western blotting (Figure 1K,L) and immunofluorescence (Figure 1N,O). To further explore the changes in intestinal permeability in PI‐IBS rats, fluorescein isothiocyanate (FITC)‐dextran was orally administered to PI‐IBS and C group rats after 8 days of modeling, and then serum FITC‐dextran levels, an indicator of intestinal permeability, were measured after 4 h. Serum FITC‐dextran levels in PI‐IBS rats were higher than those in the C group, indicating increased intestinal barrier permeability in PI‐IBS rats (Figure 1M).

Next, we explored the differences in gut microbiota composition between the C and PI‐IBS groups using 16S rRNA gene sequencing. Alpha diversity, based on Shannon (Figure S1A, Supporting Information) and Simpson indices (Figure S1B, Supporting Information), was not significantly different between the two groups. Principal coordinates analysis (PCoA) revealed that the two groups had different gut microbiota structures (Figure S1C, Supporting Information). At the phylum level, *Firmicutes*, *Bacteroidetes*, and *Proteobacteria* were the predominant phyla in fecal microbiota samples in both groups (Figure 1P). The PI‐IBS rats exhibited a higher relative abundance of *Firmicutes* and a lower abundance of *Bacteroidetes* and *Proteobacteria* (Figure S1D–F, Supporting Information). At the genus level, *Lactobacillus*, *Ruminococcaceae*, and *Lachnospiraceae* were dominant in both groups (Figure 1Q). We found that the relative abundance of *Muribaculaceae*, *Parasutterella*, *Prevotellaceae*, and *Alistipes* significantly decreased (Figure S1G,I,J,L, Supporting Information), whereas that of *Staphylococcus* sharply increased in PI‐IBS rats (Figure S1K, Supporting Information). At the species level, the abundance of *B. acidifaciens*, *Akkermansia muciniphila*, and *Pseudomonas xiamenensis* was reduced in the PI‐IBS group compared to the C group, whereas the abundance of *Staphylococcus lentus* was markedly increased (Figure 1R,S; Figure S1M–O, Supporting Information). These results suggested that 4% AA combined with bondage stress could result in increased visceral sensitivity, inflammatory reactions, intestinal mucosal barrier damage, and gut microbiota disturbances in rats.

### 2.2 Gut Microbiota Involved in the Development of PI‐IBS

To elucidate the role of gut microbiota in the pathogenesis of PI‐IBS, we pretreated rats with an antibiotic cocktail (vancomycin 0.5, ampicillin 0.5, metronidazole 1, and neomycin sulfate 1 g L^−1^; ABX; MK group) for 14 days and then rectally administered a dose of 4% AA, followed by bondage stress 7 days later (Figure S2A, Supporting Information). Although ABX treatment resulted in weight loss, it eliminated the increase in visceral sensitivity in PI‐IBS rats (M group) (Figure S2B and C, Supporting Information). Hematoxylin and eosin (H&E) staining revealed no obvious inflammatory cell infiltration or intestinal mucosal damage after ABX pretreatment (Figure S2D, Supporting Information). The levels of the serum and colon tissue proinflammatory factors IL‐1β, IL‐6, and TNF‐α were normal (Figure S2E–J, Supporting Information). Moreover, ABX pretreatment maintained normal levels of MLCK and p‐MLC proteins (Figure S2K, Supporting Information), lowered the levels of orally administered FITC‐dextran in serum (Figure S2M, Supporting Information), and stabilized the expression of the intestinal mucosal barrier proteins ZO‐1 and occludin (Figure S2L,N and O, Supporting Information).

These results indicated that ABX pretreatment exhausted the gut microbiota and eliminated the increase in visceral sensitivity, inflammatory reaction, and intestinal mucosal barrier damage, which confirmed the role of gut microbiota in the pathogenesis of PI‐IBS.

### 2.3 Rectal Administration of CGA Reduced Symptoms of PI‐IBS

**Figure 2:**
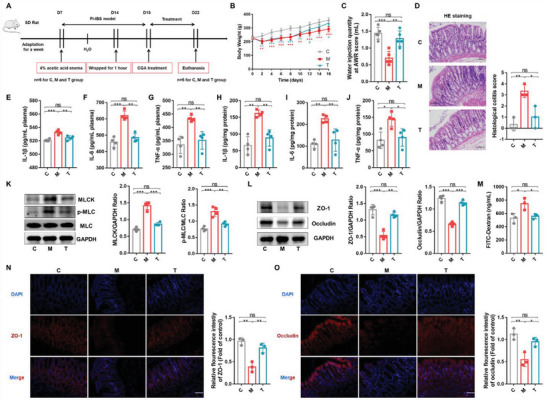
Rectal administration of CGA alleviated AA‑induced PI‐IBS in rats. A) Experimental scheme of rectal administration of CGA in PI‐IBS rats (*n* = 5). B) Changes in body weight following CGA treatment (*n* = 5). C) Water injection rate when the AWR score was 3 (*n* = 5). D) H&E staining and pathological score of colon sections (*n* = 3). E–J) Concentrations of the representative proinflammatory cytokines IL‐1β, IL‐6 and TNF‐α in rat serum and colon tissue (*n* = 4). K) Expression of key proteins of the MLCK/p‐MLC signaling pathway in rat colon tissue samples (*n* = 4). L) The expression of TJ proteins (ZO‐1, Occludin) in rat colon tissue (*n* = 4). M) Measurement of gut permeability in rats (*n* = 3). N,O) Immunofluorescence staining of TJ protein (ZO‐1, Occludin) expression in the colonic section (*n* = 3). C, control group; M, PI‐IBS rat group; T, PI‐IBS rats rectally administered CGA group. Scale bar, 100 µm. Data are presented as the mean ± SD. ns, *p* > 0.05; * *p* < 0.05; ** *p* < 0.01; ***, *p* < 0.001. CGA: chlorogenic acid.

To investigate the effect of CGA, PI‐IBS rats were rectally administered CGA for one week (dosage: 50 mg kg^−1^ of body weight daily; **Figure** **2**A). As shown in Figure 2B,C, compared with that in the PI‐IBS rats (M group), rectal administration of CGA (T group) significantly alleviated weight loss and visceral sensitivity in PI‐IBS rats. Histological analysis revealed that inflammatory cell infiltration and mucosal damage in the colon were relieved after CGA treatment (Figure 2D). To further assess the effect of CGA on systemic inflammatory responses, we measured the serum and colon tissue proinflammatory cytokine levels and observed that the levels of IL‐1β, IL‐6, and TNF‐α sharply decreased in PI‐IBS rats after CGA treatment (Figure 2E–J). Moreover, the analysis of mucosal barrier protein expression in rectal tissue samples revealed that CGA downregulated the MLCK/p‐MLC signaling pathway proteins (Figure 2K), stabilized the expression of TJ proteins (ZO‐1 and occludin) (Figure 2L, N and O), and reduced serum FITC‐dextran levels, reversing the increased intestinal permeability in PI‐IBS rats (Figure 2M). These results suggested that rectal administration of CGA could significantly alleviate visceral sensitivity, inflammatory reactions, and mucosal damage in PI‐IBS rats.

### 2.4 CGA‐Fecal Microbiota Transplantation (FMT) Alleviated PI‐IBS Symptoms in Rats

**Figure 3:**
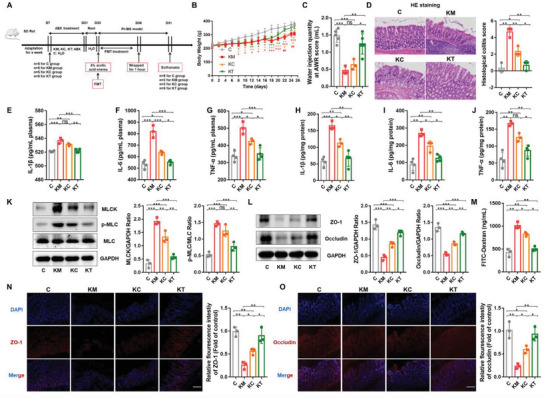
CGA‐FMT alleviated PI‐IBS symptoms in rats. A) Experimental scheme of fecal bacteria transplantation therapy in PI‐IBS rats (*n* = 5). B) Changes in body weight following FMT (*n* = 5). C) Water injection rate when the AWR score was 3 (*n* = 5). D) H&E staining and pathological score of colon sections (*n* = 3). E–J) Concentrations of the representative proinflammatory cytokines IL‐1β, IL‐6 and TNF‐α in rat serum samples and colon tissue (*n* = 3‐4). K) Expression of key proteins of the MLCK/p‐MLC signaling pathway in rat colon tissue samples (*n* = 4). L) The expression ofTJ proteins (ZO‐1, Occludin) in rat colon tissue (*n* = 4). M) Measurement of gut permeability in rats (*n* = 3). N,O) Immunofluorescence staining of TJ protein (ZO‐1, Occludin) expression in the colonic section (*n* = 3). C, control group; KM, M‐FMT group; KC, C‐FMT group; KT, T‐FMT group. Scale bar, 100 µm. Data are presented as the mean ± SD. ns, *p* > 0.05; * *p* < 0.05; ** *p* < 0.01; ***, *p* < 0.001. FMT: Fecal microbiota transplantation.

Next, we validated the effect of CGA‐exposed gut microbiota on PI‐IBS by transplanting fecal microbiota from PI‐IBS rats after rectal administration of CGA to recipient rats. We harvested fecal samples from control rats (C group), PI‐IBS rats (M group), and PI‐IBS rats after rectal administration of CGA (T group). The rats were pretreated with ABX for 14 days to deplete the abundance of gut microbiota, and after 2 days of rest, the donor rat feces were transplanted into the recipient rats (KC, KM, KT group rats) (**Figure** **3**A). The rats were monitored daily for disease status and survival. Recipients transplanted with feces from PI‐IBS rats with rectal administration of CGA (KT group) did not exhibit disease progression; two rats died in each group that received feces from only PI‐IBS (KM group) and normal rats (KC group). The KM group exhibited decreased body weight (Figure 3B) and increased visceral sensitivity (Figure 3C) compared with the KC and KT groups. H&E staining revealed that inflammatory cells infiltrated the colon tissue of the KM group. Intestinal villi were shortened, thickened, and fused, and intestinal mucosal damage was observed in the intestinal tissues of the KM rats, but these parameters were normal in the KT group (Figure 3D). Moreover, decreased serum and colon tissue levels of IL‐1β, IL‐6, and TNF‐α were observed in rats treated with CGA‐FMT (Figure 3E–J). CGA‐FMT downregulated the expression of MLCK and p‐MLC proteins (Figure 3K), upregulated the expression of ZO‐1 and occludin proteins (Figure 3L, N and O), and reduced serum FITC‐dextran levels (Figure 3M) compared to those in KM rats. Compared with the KM group, the symptoms of the KC group were alleviated after FMT, but the symptom improvement in the KT group was the most significant.

The results revealed that the transplantation of feces from CGA‐treated rats inhibited the increased visceral sensitivity, inflammatory response, and impaired intestinal mucosal barrier observed in the PI‐IBS model. Thus, the alleviating effect of CGA exerted on the PI‐IBS model may be mediated through the modulation of the gut microbiota or its metabolites.

### 2.5 CGA Rescued the Loss of Gut *B. Acidifaciens* in PI‐IBS Rats

**Figure 4:**
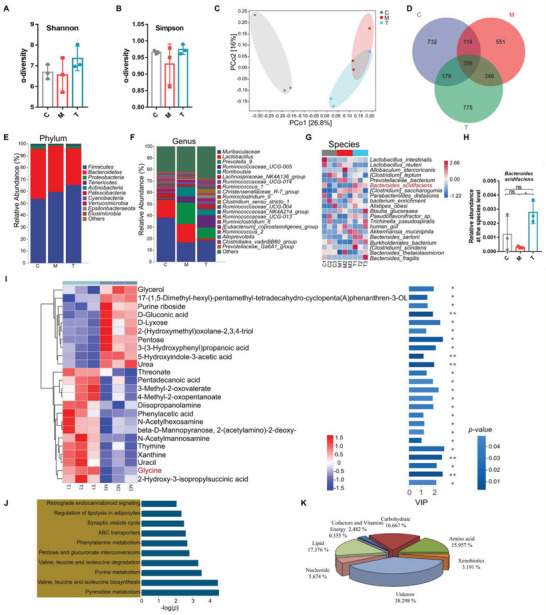
CGA increased gut *B. acidifaciens* abundance and glycine levels in PI‐IBS rats. A,B) Observed α‐diversity (Shannon and Simpson index) in control rats (C), PI‐IBS rats (M) and CGA‐rectal administration PI‐IBS rats (T) (*n* = 3). C) Observed β‐diversity (PCoA) among the three groups (*n* = 3). D) Scalar‐Venn representation among the three groups (*n* = 3). E–G) Relative abundance of the identified fecal microbiota at the (E) phylum, (F) genus, and (G) species levels as per 16S rRNA gene sequencing (*n* = 3). H) The relative abundance of *B. acidifaciens*. I) Hierarchical clustering of heatmaps of 24 significantly changed metabolites in PI‐IBS rats and CGA‐treated rats (*n* = 3). Each row in the heatmap represents a particular metabolite, and each column represents a sample. The levels of metabolites vary with color, and the relationship between color gradient and value is shown in the gradient color block. Red and blue indicate increased and decreased levels of metabolites, respectively. The metabolite cluster tree is shown on the left. Importance of metabolite variables in projection value (VIP) is shown on the right, with larger values indicating greater differences in metabolite composition between the two groups. The color of the bars indicates a statistically significant (*p* value) difference in metabolite levels between the two groups, and the smaller the *p* value, the darker the color. Glycine is labeled in red (*n* = 3). J) KEGG pathway enrichment analysis of the significantly altered metabolites; the name of each KEGG pathway is shown on the left, and the corresponding *p* value is shown on the right (*n* = 3). K) Metabolite functional classification. Each color in the pie chart represents a different classification, and its area represents the relative proportion of the metabolites (*n* = 3). C, control group; M, PI‐IBS rat group; T, PI‐IBS rats rectally administered CGA group. Data are presented as the mean ± SD. ns, *p* > 0.05; * *p* < 0.05; ** *p* < 0.01.

To explore the impact of rectal administration of CGA on the composition of the gut microbiota in PI‐IBS rats, we analyzed rat fecal microbes using 16S rRNA gene sequencing. The results revealed that CGA increased α‐diversity, but no significant differences were found in the Shannon (**Figure** **4**A) and Simpson indices (Figure 4B) among the groups. PCoA based on the unweighted UniFrac distance revealed a separation in the gut microbiota structure between normal controls and PI‐IBS rats. Moreover, a segregation was observed between the microbiota structure of CGA‐treated PI‐IBS rats and that of the PI‐IBS rats (Figure 4C). The ASV/OTU Venn diagram revealed 1429, 1417, and 1701 OTUs in the C, M, and T groups, respectively, and the percentage of common OTUs in each group was 27.92% (399/1429), 28.15% (399/1417), and 23.45% (399/1701), respectively (Figure 4D).

At the phylum level, *Firmicutes*, *Bacteroidetes*, and *Proteobacteria* were the predominant phyla in fecal microbiota samples (Figure 4E). CGA‐treated PI‐IBS rats exhibited a higher relative abundance of *Firmicutes* and *Proteobacteria*, and a lower relative abundance of *Bacteroidetes* than PI‐IBS rats (Figure S3A–C, Supporting Information). At the genus level, the fecal microbiota was dominated by *Muribaculaceae*, *Lactobacillus*, and *Prevotella* (Figure 4F). Further analysis revealed that the relative abundance of *Muribaculaceae* significantly decreased in the M and T groups (Figure S3D, Supporting Information), whereas that of *Eubacterium* and *Candidatus Saccharimonas* sharply increased in CGA‐treated rats (compared with the other two groups) (Figure S3E and F, Supporting Information). At the species level, *Lactobacillus intestinalis*, *L. reuteri*, and *Allobaculum stercoricanis* were the predominant species in the fecal microbiota (Figure 4G). Moreover, PI‐IBS rats exhibited a lower relative abundance of *B. acidifaciens* and *Parabacteroides goldsteinii* and a higher relative abundance of *Clostridium saccharogumia* and *Erysipelatoclostridium Clostridiales bacteria* after 1 week of self‐recovery than the other groups (Figure S3G and I, Supporting Information). Based on high‐throughput sequencing results (Figure 1S), we found that *B. acidifaciens* was the only species that was significantly reduced in PI‐IBS rats and sharply increased in CGA‐treated rats (Figure 4H).

These results demonstrated that the rectal administration of CGA altered the diversity of the gut microbiota, especially that of *B. acidifaciens*.

### 2.6 CGA Increased Gut Glycine Levels in PI‐IBS Rats

Next, we examined alterations in bacterial metabolite levels due to changes in microbial abundance. We performed an untargeted metabolomic analysis of fecal samples from PI‐IBS and CGA‐treated rats. We used the relative values of the metabolites under different experimental conditions (T and M groups) as metabolic levels and performed hierarchical clustering analysis. A total of 282 metabolites were analyzed, and the results are expressed as heatmaps (Figure S4A, Supporting Information). Metabolic data obtained from principal component analysis (PCA) were used to differentiate between PI‐IBS rats with or without CGA treatment. All points representing bacterial composition in fecal samples of CGA‐treated PI‐IBS rats (T) were separated from fecal samples of untreated rats (M), implying that CGA had a curative effect. However, we observed a fusion between the points in the normal control (C) and CGA‐treated groups (Figure S4B and C, Supporting Information). Metabolites with variable in projection (VIP) values > 1.0 and *p* values < 0.05 in orthogonal projections to latent structures discriminant analysis (OPLS‐DA) were selected, and 24 metabolites were identified in PI‐IBS rats with or without CGA treatment (Table S1, Supporting Information). Among these 24 metabolites, 10 were downregulated and 14 were upregulated (heatmap; Figure 4I). The changes in metabolite levels following CGA treatment are shown in column configuration based on the comparison of peak areas and FC values (Figure S5A–X, Supporting Information). Kyoto Encyclopedia of Genes and Genomes (KEGG) pathway analysis revealed that these metabolites were mainly involved in amino acid‐related metabolism, including valine, leucine, and isoleucine biosynthesis and degradation. The synaptic vesicle cycle and retrograde endocannabinoid signaling were also involved (Figure 4J and Table S2, Supporting Information). The classification of the 24 metabolites revealed that 17.376%, 16.667%, and 15.957% of the metabolites were lipids, carbohydrates, and amino acids, respectively (Figure 4K). Therefore, we further analyzed the amino acids with notably different levels between the two groups and found that the glycine content was substantially enriched in CGA‐treated rats compared with that in PI‐IBS rats (Table S1, Supporting Information).

These results highlighted that CGA‐rectal administration improved the composition of gut metabolites, especially increasing glycine levels.

### 2.7 *B. Acidifaciens* and Glycine Relieved PI‐IBS Symptoms in Rats

CGA administration modulated gut microbiota abundance (*B. acidifaciens*) and metabolite levels (glycine); therefore, we explored whether both play a role in alleviating PI‐IBS. For 1 week, we used free drinking water with 0.5% glycine and/or rectal administration of *B. acidifaciens* (1×10^9^ colony forming units; CFUs) to relieve symptoms in PI‐IBS rats (Figure S6A, Supporting Information). We found that only the *B. acidifaciens* and glycine combination treatment group (GP group) exhibited increased body weight and reduced visceral sensitivity in PI‐IBS rats (Figure S6B and C, Supporting Information). H&E staining (Figure S6D and E, Supporting Information) and enzyme‐linked immunosorbent assay (ELISA) (Figure S6F–K, Supporting Information) were used to assess the levels of colonic and systemic inflammation, respectively; they revealed a dramatic reduction in inflammation in the GP group. Evaluation of the intestinal mucosal barrier function revealed that the GP group inhibited the activation of the MLCK/p‐MLC signaling pathway (Figure S6L, Supporting Information), upregulated the expression of intestinal epithelial TJ protein, reduced serum FITC‐dextran levels, and reversed the increase in intestinal permeability of PI‐IBS rats (Figure S6M–P, Supporting Information). Thus, the combined use of probiotics and glycine was more effective for the treatment of PI‐IBS.

### 2.8 Glycine‐Induced EVs (GEVs) Restore Colonic Epithelial Barrier Function In Vitro

**Figure 5:**
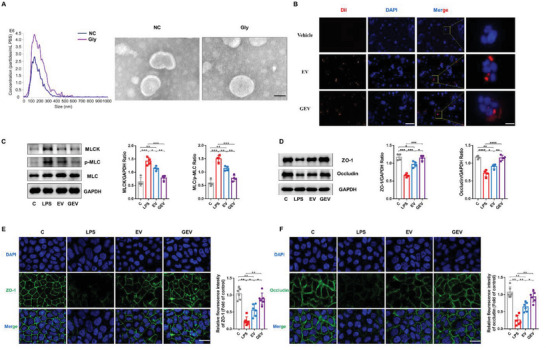
Restorative effects of glycine‐induced *B. acidifaciens* EVs on colonic epithelial barrier function in vitro. A) Morphology with or without glycine‐induced *B. acidifaciens* EVs as per TEM and size distribution analyzed by NTA. Scale bar, 50 nm. B) Dil (red)‐labeled *B. acidifaciens* EV uptake by Caco‐2 cells. Scale bars, 200 and 50 µm. C) The effect of EVs on the expression of key proteins of the MLCK/p‐MLC signaling pathway in Caco‐2 cells (*n* = 4). D–F) The effect of EVs on the expression of TJ proteins (ZO‐1, Occludin) in Caco‐2 cells was evaluated by western blotting (E, *n* = 4) and immunofluorescence (F,G) (n = 6). Scale bar, 50 µm. C, control group; LPS, LPS‐induced group; EV, EV‐treated group; GEV, GEV‐treated group; EVs, extracellular vesicles; GEV, glycine‐induced *B. acidifaciens* extracellular vesicles; EV, without induction *B. acidifaciens* extracellular vesicles. Data are presented as the mean ± SD. ns, *p* > 0.05; *, *p* < 0.05; **, *p* < 0.01; ***, *p* < 0.001; ****, *p* < 0.0001.

Next, we investigated the mode of action of glycine and *B. acidifaciens*. We speculated that glycine plays a role in alleviating PI‐IBS symptoms by promoting *B. acidifaciens*‐EV formation or altering the EV protein profile. First, we cultured *B. acidifaciens* with different concentrations of glycine (0%, 0.5%, 1%, 1.5%, and 2%). Glycine at final concentrations of 0.5%, 1%, 1.5%, and 2.0% inhibited bacterial growth in a dose‐dependent manner (Figure S7A, Supporting Information). A previous study reported that glycine can promote the production of EVs from *Escherichia coli* (*E. coli*) strain Nissle 1917.^[^ ^20^ ^]^ Next, we isolated and purified EVs from *B. acidifaciens* to explore the effects of glycine on EVs. Transmission electron microscopy (TEM) of GEVs and noninduced EVs revealed saucer‐like vesicle morphology. Nanoparticle tracking analysis (NTA) showed that the diameter of the EVs was 133.5 nm. The diameter of GEVs changed, and vesicles with diameters of 133.6 and 188.4 nm were observed (**Figure** **5**A). Coomassie brilliant blue (CBB) staining (Figure S7B, Supporting Information) revealed differences between the protein spectra of the two groups. For example, compared to that of GEVs, the protein band density at 110 kDa was significantly higher in EVs.

To further investigate the role of EVs in microbe–host interactions, we examined whether EVs could be taken up by intestinal epithelial cells in vitro. After coincubation of EVs with Caco‐2 cells for 6 h (Figure 5B), the red‐Dil signal was absorbed by the cells and accumulated in the perinuclear region, suggesting that EVs may have regulatory effects on cell function. We used a cell counting kit‐8 (CCK‐8) to examine the repair effect on LPS‐induced Caco‐2 cell injury. After LPS (20 µg mL^−1^) stimulation of Caco‐2 cells for 24 h, 5, 10, 20, and 30 µg mL^−1^ EVs or GEVs were added and incubated with the cells for another 24 h. The results showed that the 10 µg mL^−1^ dose group exhibited higher cell viability in both groups, especially in the GEV group (Figure S7C and D, Supporting Information). Furthermore, EVs exhibited profound barrier repair effects in LPS‐treated cells. GEVs had significantly higher values than EVs, the expression of MLCK and p‐MLC proteins was downregulated (Figure 5C), and the levels of intercellular TJ proteins (ZO‐1 and occludin) were much higher (Figure 5D). The same results were obtained for these proteins in Caco‐2 cells using immunofluorescence (Figure 5E and F).

Therefore, glycine can induce the formation of *B. acidifaciens* EVs and alter their protein profile. Furthermore, both EVs and GEVs can enter Caco‐2 cells, but GEVs can more significantly promote the repair of intestinal epithelial barrier function than EVs.

### 2.9 GEVs Ameliorated Symptoms in PI‐IBS Rats

**Figure 6:**
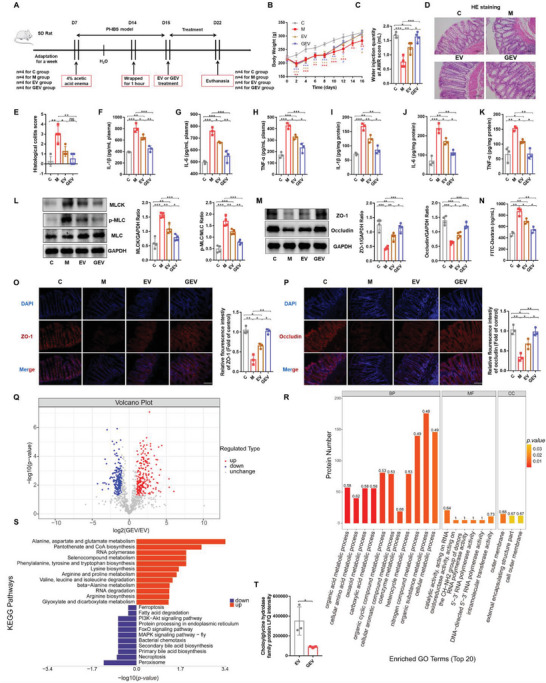
Glycine ameliorates PI‐IBS symptoms in rats by altering the protein profile of *B. acidifaciens* EVs. A) Experimental scheme of rectal administration of EVs in PI‐IBS rats (*n* = 4). B) Changes in body weight following EV treatment (*n* = 4). C) Water injection rate when the AWR score was 3 following treatments with EVs (*n* = 4). D,E) H&E staining and pathological score of colon sections (*n* = 4). F–K) Concentrations of the representative proinflammatory cytokines IL‐1β, IL‐6, and TNF‐α in rat serum samples and colon tissue (*n* = 3). L) Expression of key proteins of the MLCK/p‐MLC signaling pathway in rat colon tissue (*n* = 4). M) The expression of TJ proteins (ZO‐1, Occludin) in rat colon tissue (*n* = 4). N) Measurement of gut permeability in rats (*n* = 3). O,P) Immunofluorescence staining of TJ protein (ZO‐1, Occludin) expression in the colonic section (*n* = 3). Q) Volcano plot showing the number of proteins differentially expressed between GEVs and EVs, with a cutoff of *p* <0.05 and |fold change| >2. R) GO classification (biological process, molecular function, and cellular components) of all differentially expressed proteins in glycine‐induced *B. acidifaciens* EVs and the top 20 enriched GO terms. S) KEGG pathway enrichment analysis of the differentially expressed proteins in GEVs compared to their expression in EVs. T) Choloylglycine hydrolase family protein LFQ intensity (*n* = 3). C, control group; M, PI‐IBS rat group; EV, PI‐IBS rats rectally administered EV group; GEV, PI‐IBS rats rectally administered GEV group. Scale bar, 100 µm. Data are presented as the mean ± SD. ns, *p* > 0.05; * *p* < 0.05; ** *p* < 0.01; ***; *p* < 0.001.

Next, we determined the effect of GEVs and EVs on PI‐IBS rats via the rectal administration of EVs (50 µg day^−1^) or GEVs (50 µg day^−1^) (**Figure** **6**A). As shown in Figure 6B,C, compared with the EV group, GEV significantly alleviated body weight loss and enhanced visceral sensitivity in PI‐IBS rats. Consistently, rectal administration of EVs or GEVs significantly reversed intestinal mucosal inflammation and pathological changes in the intestinal villi of PI‐IBS rats (Figure 6D,E). Moreover, GEVs exhibited a more significant effect on reducing the levels of proinflammatory factors (IL‐1β, IL‐6, and TNF‐α) than EVs in serum and colon tissue (Figure 6F–K). Analysis of the MLCK/p‐MLC signaling pathway proteins (Figure 6L), intestinal interepithelial TJ proteins (ZO‐1 and occludin; Figure 6M, O and P) and serum FITC‐dextran levels (Figure 6N) revealed that GEVs had a more obvious effect in enhancing mucosal barrier integrity. In vitro fluorescence imaging revealed increased fluorescence signals not only in the rat liver and kidneys but also in the brain tissue after rectal administration of Dil‐labeled EVs or GEVs for 3 h (Figure S8A, Supporting Information). Figure S8B (Supporting Information) showed the presence of PKH26‐labeled EVs or GEVs in the liver, kidneys, and brain in mice, suggesting that these bacterial EVs could enter the host brain tissue to play a regulatory role. As a control, the gram‐positive bacterium *Lactobacillus salivarius* did not exhibit a similar tissue distribution (Figure S9, Supporting Information).

### 2.10 Glycine Altered the Protein Profile of *B. Acidifaciens* EVs

Proteins in *B. acidifaciens* EVs, with or without glycine induction, were characterized and quantified using label‐free proteomic analysis. We identified 956 proteins in the EVs and GEVs, of which 947 were quantified (Figure S10A, Supporting Information). We found that 0.5% glycine supplementation resulted in 208 upregulated differentially expressed proteins (DEPs) and 183 downregulated DEPs (Figure 6Q). Venn diagram analysis showed that there were fewer unique differentially expressed proteins identified in GEVs than in EVs (Figure S10B, Supporting Information). The DEPs of the two groups (EV and GEV) were grouped and classified using hierarchical clustering analysis (heatmap; Figure S10C, Supporting Information). Domain prediction and enrichment analysis of DEPs revealed that the TonB‐dependent reporter and TonB‐dependent reporter plug domains were the most significantly enriched domains (Figure S10D and E, Supporting Information). To clearly identify glycine‐induced biological alterations in *B. acidifaciens* EVs, the DEPs were classified into three categories (biological process, BP; molecular function, MF; cellular component, CC) based on Gene Ontology (GO) analysis (Figure 6R), and the top 20 terms are displayed in the figure. The most significant proportion of DEPs was concentrated in bacterial biological processes, including nitrogen compound metabolic processes, organic substance metabolic processes, and cellular metabolic processes. Furthermore, KEGG pathway analysis revealed that the DEPs from GEVs mainly upregulated some amino acid metabolism‐related pathways, including alanine, aspartate and glutamate metabolism, pantothenate and CoA biosynthesis, and phenylalanine, tyrosine and tryptophan biosynthesis, suggesting that glycine most likely affected EV metabolism, especially amino acid and energy metabolism. Moreover, glycine downregulated the PI3K/Akt signaling pathway, FoxO signaling pathway and MAPK signaling pathway‐fly, which are common signaling pathways in EVs, and affected bile acid metabolism, including secondary bile acid biosynthesis and primary bile acid biosynthesis, as shown by the downregulation of choloylglycine hydrolase family protein (Figure 6S and T). Thus, GEVs undergo dramatic changes in protein composition compared to uninduced EVs, ultimately altering their biological functions.

## 3 Discussion

**Figure 7:**
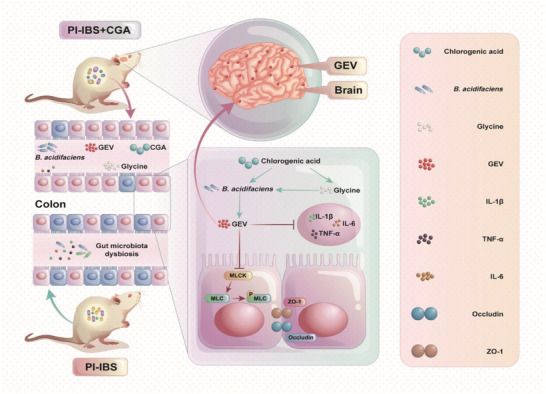
A schematic diagram summarizing the main findings of this study.

In this study, we explored the relationship between gut microbes and PI‐IBS and confirmed the beneficial role of CGA in PI‐IBS. Moreover, we found that *B. acidifaciens* and glycine could be responsible for the alleviating effects of CGA on PI‐IBS symptoms. Finally, we demonstrated that glycine‐induced *B. acidifaciens* EVs can alleviate PI‐IBS symptoms and enter the brain tissue to play a potential regulatory role (**Figure** **7**).

The gut microbiota has a remarkable ability to resist environmental disturbances and maintain its structure and function.^[^ ^21^ ^]^ Ecosystem restoration of the gut occurs after it has recovered from infection. However, PI‐IBS patients may not be able to restore their gut microbial ecosystem because of host factors.^[^ ^5b^ ^]^ Therefore, we first investigated the specific role of the gut microbiota in the PI‐IBS rat model (Figure 1). We observed PI‐IBS symptoms in model rats induced by 4% AA and bondage stress, which were consistent with those observed in previous studies, including body weight loss, visceral hypersensitivity, systemic and intestinal inflammation, and impaired intestinal mucosal barrier function.^[^ ^22^ ^]^ Systemic inflammation was manifested by elevated levels of the serum proinflammatory factors IL‐1β, IL‐6, and TNF‐α, which have been reported in several clinical and basic studies.^[^ ^23^ ^]^ Moreover, intestinal mucosal inflammation and impaired barrier function were observed in PI‐IBS rats, which is consistent with previous similar studies.^[^ ^22^ ^]^ The intestinal mucosal barrier is essential for defense against antigen invasion, maintaining homeostasis, sequestering luminal contents, and preventing intestinal microbial migration.^[^ ^24^ ^]^ The TJ proteins between intestinal epithelial cells, including the transmembrane protein occludin and cytoplasmic protein ZO‐1, are an important part of the intestinal mucosal barrier.^[^ ^25^ ^]^ Moreover, the MLCK/p‐MLC pathway has profound effects on intestinal epithelial barrier dysfunction through TJ structural perturbations. MLC is activated and phosphorylated by MLCK, which can induce the breakdown of the TJ structural protein ZO‐1 under specific pathological conditions and increase intestinal permeability.^[^ ^26^ ^]^ Our previous study showed that the activation of the MLCK/p‐MLC signaling pathway could lead to the degradation of intestinal epithelial TJ proteins and increase intestinal permeability.^[^ ^27^ ^]^ Qiang et al. demonstrated that the levels of MLCK increased and those of ZO‐1 and occludin decreased in PI‐IBS rats.^[^ ^22^ ^]^

Moreover, 16S rRNA high‐throughput sequencing revealed dysbiosis in PI‐IBS rats (Figure 1; Figure S1, Supporting Information). PCoA revealed that the gut microbiota structure of PI‐IBS rats was significantly altered, with segregation from that of normal rats. Further analysis of gut microbiota composition in the rat model revealed a reduction in the abundance of *Alistipes*, which was consistent with the results of a previous study that patients with PI‐IBS exhibited a lower abundance of *Alistipes putredinis*.^[^ ^28^ ^]^ Previous studies have reported that supplementation with soluble high‐fiber diets upregulates the relative abundance of *B. acidifaciens*, which may improve intestinal immune function, thereby preventing the entry of enteric pathogens and reducing the incidence of inflammatory bowel disease.^[^ ^16^ ^]^ A recent study elucidated *B. acidifaciens* as a vital gut bacterial species for liver homeostasis, as it can reduce hepatocyte apoptosis in a CD95‐dependent manner and ameliorate liver injury.^[^ ^15^ ^]^ *Akk* has excellent SCFA production capacity, and previous studies have reported that the relative abundance of *Akk* is positively correlated with the production of SCFAs and negatively correlated with the production of pro‐inflammatory cytokines.^[^ ^29^ ^]^ Coagulase‐negative *Staphylococcus* (CoNS) are opportunistic pathogens that are reservoirs of antibiotic resistance genes. *Staphylococcus lentus* (*S. lentus*) is a CoNS that was significantly enriched in rats with PI‐IBS in a previous study.^[^ ^30^ ^]^ Next, we explored the role of gut microbiota in the occurrence of PI‐IBS (Figure S2, Supporting Information) and observed that early administration of the antibiotic cocktail resulted in no PI‐IBS‐like symptoms in addition to weight loss in rats. The likely reason for the weight loss is that the use of antibiotics depleted the abundance of gut microbiota, leading to poor nutrient breakdown and absorption. These results indicate that PI‐IBS rats exhibit dysbiosis and that the gut microbiota plays a role in the pathogenesis of the disease.

Previous studies have shown that CGA can regulate the gut microbiota, rescue colitis, and relieve neuropsychiatric diseases, such as anxiety.^[^ ^31^ ^]^ We demonstrated that CGA alleviated PI‐IBS symptoms in rats (Figure 2). Rectal administration of CGA reduced systemic inflammation, attenuated intestinal sensitivity and pathology, and reduced colon tissue inflammation and serum levels of inflammatory cytokines. A previous study on the alleviation of DSS‐induced colitis by CGA reported that CGA can downregulate the levels of the proinflammatory factors IL‐1β and TNF‐α and inhibit colonic infiltration of inflammatory cells, such as neutrophils.^[^ ^32^ ^]^ Moreover, therapeutic rectal CGA administration significantly reduced intestinal permeability in PI‐IBS rats. Wang et al. reported that CGA regulates the gut microbiota, increases the expression of intestinal ZO‐1 and occludin proteins, reduces the levels of inflammatory factors (IL‐1β and IL‐6), and maintains intestinal homeostasis.^[^ ^33^ ^]^ We elucidated the efficacy of rectal administration of CGA in rats with PI‐IBS, but the specific mechanism remains to be explored.

To elucidate the role of CGA in PI‐IBS rats in regulating the gut microenvironment, we performed FMT (Figure 3). The KC, KM, and KT groups were transplanted with fecal bacteria from the normal control (C), PI‐IBS (M), and CGA treatment (T) groups at the same time as in the AA‐induced PI‐IBS model, respectively. CGA‐FMT reversed the symptoms of PI‐IBS induced by AA and bondage stress, exhibiting a trend toward normal control rats, whereas PI‐IBS‐FMT rats exhibited a worse condition than C‐FMT rats. A possible explanation could be that the feces of the rats in the CGA treatment group contained substances that relieved PI‐IBS symptoms. Based on these results, we hypothesized that CGA‐mediated gut microbes and their metabolites were responsible for their mitigating effects.

Furthermore, we explored the differences between the feces of rats rectally administered CGA and the other two groups (C and M). The results of the α‐diversity analysis showed that CGA supplementation increased the diversity of the gut microbiota, and PCoA revealed that there were differences in the microbiota structure among the normal control, PI‐IBS, and CGA treatment groups. Moreover, the microbiota structure of the CGA group gradually approached that of the normal group. Consistent with the results obtained in the previous phase of the experiment (Figure S1, Supporting Information), the relative abundance of *B. acidifaciens* remained low after 1 week of self‐healing in PI‐IBS rats, which was reversed by rectal administration of CGA. Additionally, fecal metabolome analysis revealed that glycine was the most significantly different metabolite among metabolites with elevated levels (Figure 4; Figure S5, Supporting Information). *Yulong Yin* et al. also reported that supplementation with CGA could increase glycine levels in rats. The increase in glycine levels may be due to the ability of CGA to promote endogenous glycine synthesis in mammalian cells and intestinal microorganisms, as well as enhance the absorption of glycine by the intestinal lumen.^[^ ^34^ ^]^ Previous studies have shown that glycine can prevent experimental colitis in rats by reducing the intestinal inflammatory response and repair intestinal mucosal damage by upregulating the expression of TJ proteins.^[^ ^35^ ^]^ According to a study by Herbert Y. Meltzer et al., plasma glycine levels in patients with schizophrenia and major depression were lower than those in the control group.^[^ ^36^ ^]^ Furthermore, we observed that although *B. acidifaciens* and glycine alleviated PI‐IBS symptoms, the combined treatment was more effective than the individual treatments (Figure S6, Supporting Information). These results confirmed that the effect of CGA on PI‐IBS occurred through the regulation of the gut microenvironment (including gut microbiota and metabolites). However, the regulatory role of the mediators in the gut microenvironment needs to be further explored.

The secretion of bacterial EVs is a mechanism of information exchange between the gut microbiota and host cells. EVs selectively carry various molecules (e.g., nucleic acids and proteins) from their bacteria, which alters the function of the recipient cells.^[^ ^37^ ^]^ Previous studies have reported that glycine can promote the quasilysis of *E. coli* strain *Nissle* 1917, increase the number of EVs, enlarge particle sizes, and alter protein profiles.^[^ ^20^ ^]^ Therefore, we investigated whether glycine could affect the formation of *B. acidifaciens* EVs and observed that glycine induction significantly altered *B. acidifaciens* EVs, including their number, size, and protein expression profile (Figure 5; Figure S7, Supporting Information). Furthermore, GEVs exhibited better barrier repair ability in vitro and a more prominent ability to alleviate PI‐IBS symptoms in vivo; they even entered the brain to play a potential regulatory role (Figure 6; Figure S8, Supporting Information). Compared with nonglycine‐induced EVs, GEVs were enriched with a variety of functional proteins, which may directly promote intestinal epithelial cell repair and alleviate inflammation. However, the proteins that mediate the benefits of GEVs in PI‐IBS require further investigation. De Vos et al. reported decreased amino acid synthesis, disruption of cell junction integrity, and inflammatory responses in patients with PI‐IBS, suggesting impaired epithelial barrier function.^[^ ^38^ ^]^ Metabolomic analysis after rectal CGA‐FMT and proteomics analysis of GEVs revealed that amino acid metabolism was enriched by the DEPs.^[^ ^38^ ^]^ Anxiety caused by brain injury may be related to the activation of the PI3K/AKT/FoxO1 pathway.^[^ ^39^ ^]^ Moreover, mitogen‐activated protein kinases (MAPKs) are activated in response to various stress stimuli and play a vital role in antidepressant and anxiety‐like behaviors by inhibiting the activation of the MAPK signaling pathways.^[^ ^40^ ^]^ We found that there were significant differences in choloylglycine hydrolase family proteins, which are enzymes related to bile acid synthesis.^[^ ^41^ ^]^ Bile acids can affect visceral sensitivity, and previous studies have suggested that bile acid absorption is abnormal in patients with diarrhea‐type IBS.^[^ ^42^ ^]^ Excessive concentrations of bile acids in the colon can cause watery stools and abnormal defecation habits by affecting colonic motility and secretory functions.^[^ ^5a^ ^]^ In the current study, proteomic analysis of GEVs revealed the downregulation of the abovementioned signaling pathways and inhibition of bile acid synthesis, which may be related to the alleviation of PI‐IBS symptoms; however, the specific mechanism requires further exploration.

## 4 Experimental Section

### Ethics Statement

Animal care and experimental procedures were approved by the Laboratory Animal Ethics Committee of Nanchang University (NCUFII‐2021411).

### Animal Experiments

We used 8‐week‐old male Sprague Dawley rats in this study, which were provided by Changsha Tianqin Biotechnology Co., Ltd. (Hunan, China). The rats were maintained under standard temperature, humidity, and light conditions (room temperature: 22 °C, humidity: 55%–60%, and light/dark cycles for 12 h) and were allowed free access to food and water.^[^ ^43^ ^]^ Commercial rat chow (Changsha Tianqin Biotechnology Co., Ltd.) and water were autoclaved prior to use.

After 1 week of acclimation, as described in previous studies, the rats were anesthetized by ether inhalation, 1 mL of 4% AA was slowly injected into their colon (8 cm near the anus) for 30 s, and the colon was flushed with phosphate‐buffered saline (PBS); after 7 days, the rats were confined in a fixed transparent box for 1 h for bondage stress to induce PI‐IBS.^[^ ^22^ ^]^ To explore the occurrence of dysbiosis in PI‐IBS rats, feces from normal or PI‐IBS rats were collected 8 days after AA injection for 16S rRNA gene sequencing. To further determine the role of microbes in the PI‐IBS model, the rats were administered an antibiotic cocktail (vancomycin 0.5, ampicillin 0.5, metronidazole 1, and neomycin sulfate 1 g L^−1^; ABX) for 14 days before AA induction.^[^ ^44^ ^]^ To assess the therapeutic effect of CGA on PI‐IBS, CGA (50 mg kg^−1^) or an equal volume of sterile water was rectally administered to AA‐treated rats for 1 week. To assess the effect of FMT on PI‐IBS, rat feces were collected 1 week after CGA or sterile water treatment to prepare normal, CGA‐, or sterile water‐treated rat fecal microbiota suspensions (KC, KT, KM). Fresh feces from different donors were diluted with PBS, thoroughly mixed, suspended (100 mg feces mL^−1^), and centrifuged at 500 × *g* for 1 min to collect the supernatant.^[^ ^45^ ^]^ The number of bacteria was determined using the colony counting method. After the recipient rats were microbially depleted for 14 days and rested for 2 days, FMT (1×10^9^ CFUs, 1 week) was performed at the same time as AA induction. After 14 days of ABX treatment, fecal samples were placed on brain‐heart infusion broth (BHI) agar plates for 96 h of anaerobic incubation to ensure fecal microbiota failure.^[^ ^46^ ^]^ To explore the role of *B. acidifaciens* or glycine in PI‐IBS, PI‐IBS rats were rectally administered *B. acidifaciens* (1×10^9^ CFUs), free drinking 0.5% glycine water, or a combination of both (P, G, or GP) for 1 week. To evaluate the influence of *B. acidifaciens* EVs or 0.5% GEVs on PI‐IBS, AA‐treated mice were rectally administered EVs (50 µg mL^−1^), GEVs (50 µg mL^−1^), or an equal volume of PBS for 1 week.^[^ ^47^ ^]^ Fecal samples of the rats were collected for 16S rRNA gene sequencing or untargeted metabolomics analyses. The serum samples were centrifuged at 1000 × *g* at 4 °C for 15 min and stored at 80 °C for further analysis. The success of the PI‐IBS model was measured using the abdominal withdrawal reflex (AWR).

### AWR

A latex double‐lumen catheter connected to a balloon was used to detect AWR. After ether inhalation, the catheter was inserted into the rectum of the rats (8 cm from the anus), and rectal distention was maintained by water injection. The rats were placed in a transparent box where they could move only up and down. The AWR response was observed and evaluated according to the following scale: 0, no response; 1, head moves briefly and then stays still; 2, abdominal muscle contraction; 3, abdomen is lifted; and 4, body arch and improvement of pelvic structure. In this study, when the AWR score was 3, the water injection rate was recorded.^[^ ^48^ ^]^

### Intestinal Permeability Assays

Barrier function was assessed by measuring the in vivo paracellular permeability of fluorescein isothiocyanate‐labeled dextran (FD4; Sigma‒Aldrich). Briefly, rats were orally administered 1 mL of FITC‐dextran (25 mg mL^−1^). Rat serum was obtained after 4 hours. Serum dextran concentration was calculated by comparing serial dilutions of the sample with known standard solutions using a multifunctional microplate reader (Thermo Fisher, Varioskan Flash) with excitation at 488 nm and emission at 525 nm.^[^ ^46a^ ^]^

### Culture of Bacteria

*B. acidifaciens* (BNCC353574, BeNa, Henan, China) was cultured on Columbia agar with 5% goat blood (Cat# TX0020; Solarbio, Inc., China) and fastidious anaerobe broth (FAB, Cat# LA4550; Solarbio, Inc.) with or without glycine under anaerobic conditions at 37 °C; anaerobic atmosphere was confirmed by an anaerobic indicator (Cat# C22; Mitsubishi Gas Chemical, Japan) for 48–72 h. *L. salivarius* (BNCC138618, BeNa, Henan, China) was cultured in De Man Rogosa and Sharpe (MRS; HB0384‑1; Qingdao Hope Bio‐Technology, Co., Ltd.) broth for 24 h in an incubator at 37 °C under aerobic conditions. The bacterial concentration was calculated by measuring the absorbance at 600 nm or by counting the number of CFUs of bacteria after plating on Columbia agar containing 5% goat blood or MRS agar plates.^[^ ^18^ ^]^

### Isolation, Identification, and Labeling of EVs from *B. Acidifaciens* and *L. Salivarius*

EVs were isolated from *B. acidifaciens* cultured in FAB for 4 days. LSEVs were isolated from *L. salivarius* cultured in MRS for 24 h. Then, the conditioned medium of this bacterium was obtained, centrifuged twice at 5000 ×*g* at 4 °C for 30 min, filtered with a 0.45 µm filter to remove large polymer particles, and then filtered through a 0.22 µm filter to remove residual bacteria. The supernatant was concentrated by transfer to 100 kDa Amicon Ultra15 filters, vortexed, aerated for 20 min, and then ultracentrifuged at 150 000 × *g* for 90 min. EVs were purified by OptiPrep gradient density centrifugation, and 219 µL of EVs were mixed with 2,291 µL of OptiPrep solution (60% w/v iodixanol; Cat# D1556, Sigma–Aldrich, USA), and then 1 mL of OptiPrep solution (10%–50%, v/v) with different concentrations was added in turn from high concentration to low concentration in a 12.5 mL polyallomer Beckman Coulter tube. The sample was covered and centrifuged at 100 000 × *g* and 4 °C for 18 h. The layers rich in EVs were diluted, washed with PBS, and centrifuged at 150 000 × *g* at 4 °C for 2 h to obtain purified EVs. Plate coating ensured that the purified EVs were free of bacterial contamination. The size and number of EVs were tested using NTA (Particle Metrix Zetaview, Germany). The protein content of the EV samples was evaluated using a BCA protein assay kit (Cat# PC0020; Solarbio, Inc.). EV samples were stored at −80 °C until use. The purified EV sample was mixed with Dil (Cat# D8700; Solarbio, Inc.) solution (5 µM) in equal volume, incubated in the dark at 37 °C for 30 min, centrifuged at 150 000 × *g* at 4 °C for 1.5 h, washed three times, and resuspended in PBS.^[^ ^17^, ^18^, ^19^ ^]^

### TEM

TEM was used to observe and analyze the EVs. Briefly, the EV preparation was placed on a copper net and fixed using glutaraldehyde. After washing with ddH_2_O, the copper nets were placed in drops of uranyl oxalate and methylcellulose. Finally, the samples were air dried for 5–10 min and visualized by TEM.^[^ ^49^ ^]^

### Cell Culture

Caco‐2 cells (BNCC350769, BeNa, Henan, China) were cultured in high glucose DMEM (Cat# 11 995; Solarbio, Inc.) containing 1% penicillin–streptomycin and 10% fetal bovine serum (FBS; Cat# 1611A; Bovogen, Australia) in a humid environment at 37 °C and 5% CO_2_.

### EV Uptake Assay

To evaluate the ability of Caco‐2 cells to uptake EVs, Dil‐labeled EVs were cocultured with the cells for 6 h, washed with PBS, and fixed with 4% paraformaldehyde (Cat# P1110; Solarbio, Inc.) for 30 min. After washing with PBS three times, DAPI (Cat# C0065; Solarbio, Inc.) was used to stain the nuclei, and fluorescence microscopy was performed.^[^ ^17^ ^]^

### Cell Viability Assay

Caco‐2 cells were seeded in 96‐well plates at a density of 2 × 10^4^/well and cultured overnight. The cells were stimulated with 20 µg mL^−1^ LPS (Cat# L8880; Solarbio, Inc.) for 24 h and then treated with EVs at doses of 5, 10, and 20 µg mL^−1^ for 24 h. Then, 10 µL of CCK‐8 (Cat# BA00208; Bioss, Inc., China) solution was added to each plate, and the plates were incubated for another 2 h. Finally, the absorbance was measured at a wavelength of 450 nm using a microplate reader.^[^ ^50^ ^]^

### Cell Immunofluorescence Assay

Caco‐2 cells were grown in confocal dishes, treated with LPS and EVs, as described above, fixed with 4% paraformaldehyde for 30 min, and blocked with 5% bovine serum albumin (BSA; Cat# A8010; Solarbio, Inc.) for 2 h at 37 °C. After washing the cells with PBS, primary antibodies rabbit anti‐occludin (occludin; 1:400; Cat# 27260‐1‐AP; Proteintech Group, Inc., China) and rabbit anti‐ZO‐1 (1: 500; Cat# 21773‐1‐AP; Proteintech Group, Inc.) were added, and the cells were incubated at 4 °C overnight. They were washed three times with PBS and incubated with CoraLite488‐conjugated goat anti‐rabbit (1:100; Cat # SA00013‐2; Proteintech Group, Inc.) at room temperature for 2 h. After staining the nuclei with DAPI, the cells were visualized under a confocal microscope.^[^ ^51^ ^]^

### SDS‒PAGE and Western Blotting

Proteins from EVs, whole‐cell, and intestinal samples were extracted using cell lysis buffer supplemented with a protease inhibitor cocktail (Cat# R0010; Solarbio, Inc.) and 1 mM phenylmethanesulfonyl fluoride (PMSF) (Cat# P0100; Solarbio, Inc.). Protein content was measured using a BCA protein assay kit (Cat# PC0020; Solarbio, Inc.). EV proteins were separated by Tris‐glycine SDS‒PAGE (12.5%) and stained with CBB (Cat# G4540; Solarbio, Inc.). Whole‐cell and intestinal‐tissue proteins were detached using 6%–12% polyacrylamide resolving gels, transferred onto polyvinylidene fluoride (PVDF) membranes, and blocked with 5% nonfat milk (Cat# P1622‐1; Beijing Applygen Technologies, Inc., China) for 90 min at room temperature. The membranes were incubated overnight at 4 °C with the following primary antibodies: rabbit anti‐MYLK (MLCK; Cat# 21642‐1‐AP; Proteintech Group, Inc.), rabbit anti‐myosin light chain 2 (MLC; Cat# 10906‐1‐AP; Proteintech Group, Inc.), rabbit anti‐phosphorylated myosin light chain 2 (p‐MLC; Cat# PA5‐104265; Thermo Fisher; USA), rabbit anti‐ZO‐1, rabbit anti‐occludin, and mouse anti‐GAPDH (GAPDH; Cat# 60004‐1‐Ig; Proteintech Group, Inc.). After washing the membranes three times with TBST buffer, they were incubated with horseradish peroxidase (HRP)‐conjugated goat anti‐rabbit secondary antibody (1:5000; Cat# PR30011; Cat# 60004‐1‐Ig; Proteintech Group, Inc.) or goat anti‐mouse secondary antibody (Cat# PR30012; Cat# 60004‐1‐Ig; Proteintech Group, Inc.) for 60 min at room temperature. Finally, the proteins were visualized using super enhanced chemiluminescence reagent (Cat# PA112; Tiangen Biotech Co., Ltd., China).^[^ ^20^, ^27^ ^]^

### H&E Staining and Immunofluorescence

Colorectal tissues were fixed with 4% paraformaldehyde, dehydrated with ethanol, and embedded in paraffin. The paraffin‐embedded tissues were cut into thick slices (5–6 µm), and the samples were dewaxed using xylene and degraded ethanol. Finally, the sections were subjected to H&E staining and immunofluorescence (ZO‐1; 1: 100; Cat# 21773‐1‐AP; Proteintech Group, Inc. and occludin; 1:100; Cat# 27260‐1‐AP; Proteintech Group, Inc., China), and morphological changes and protein expression were observed.^[^ ^43^ ^]^ The histological scores were determined as previously described.^[^ ^52^ ^]^ The ZO‐1 and occludin fluorescence signal intensities were measured by ImageJ 1.46r.

### ELISA

Rat serum samples were obtained from the venous blood, which was collected from the inferior vena cava of the rats and centrifuged at 1000 × *g* for 15 min at 4 °C. Intestinal tissue protein was extracted as described above, and protein quantification was performed using a BCA kit. The concentrations of cytokines IL‐1β (Cat# ERC007.96, QuantiCyto, China), IL‐6 (Cat# ERC003.96, QuantiCyto), and TNF‐α (Cat# ERC102a.96, QuantiCyto) in the serum and intestinal tissue samples were quantified using an ELISA kit, according to the manufacturer's protocol.^[^ ^43^ ^]^

### In Vivo Tissue Distribution of EVs

GEVs or EVs were labeled with Dil as previously described and resuspended in PBS. Rats were fasted for 24 h and treated rectally. The control rats were treated with an equal volume of PBS. After 3 h, all rats were sacrificed, and the intestine, liver, kidney, spleen, lung, heart, and brain were collected and photographed with IVIS Lumina (Tanon ABL X6, Shanghai, China).^[^ ^17^ ^]^

EVs were labeled with PKH26 (Cat# D0030; Solarbio, Inc.) and used to test the tissue distribution of these EVs. After removal of the excess dye by the procedure described above, 1 mL of PBS with or without EVs was administered rectally to fasted rats. After 3 h, brain, kidney, and liver tissues were collected and exposed to liquid nitrogen for 15 s before being transferred to −80 °C storage for preservation. The sections were embedded in OCT embedding medium (Cat# G6059‐110ML; Servicebio, Inc.) and cut into sections with a thickness of 4–5 µm. The nuclei were stained with DAPI. Images were obtained using a fluorescence microscope, and the intensity of the fluorescence signal was measured by ImageJ.^[^ ^18^, ^53^ ^]^

### DNA Extraction and 16S rRNA Gene Sequencing

Total microbial genomic DNA from rat fecal samples was extracted using genomic DNA kits (Tiangen Biotech Co., Ltd.) combined with the bead‐beating method. The DNA samples were sent to Personalbio Biotechnology Co., Ltd. (Shanghai, China), and the composition and relative abundance of the gut microbiota were analyzed by high‐throughput 16S rRNA gene sequencing. The variable V4 region of the 16S rRNA gene of each sample was amplified using 515F/806R primers (515F, 5′‐AYTGGGYDTAAAGNG‐3′; 806R, 5′‐TACNVGGGTATCTAATCC‐3′), and the PCR products were subjected to paired‐end sequencing of the community DNA fragments using the Illumina HiSeq 2000 platform (Illumina, Inc., San Diego, CA, USA). High‐quality sequences were clustered at a 97% similarity level using DADA2 sequence denoising (QIIME2 2019.4) and Vsearch clustering (Vsearch v2.13.4_linux_x86_64; cutadapt v2.3), and representative sequences and OTUs were output. Subsequently, singleton OTUs (OTUs with an abundance of 1 in all samples) and their representative sequences were removed from the OTUs. Functional gene correction (FrameBot v1.2), high‐quality sequence length distribution statistics, and reference sequence databases were also used to classify species by phylum, class, order, family, genus, and species (GenBank accession number PRJNA961049).^[^ ^54^ ^]^

### Untargeted Metabolomic Analyses

Samples of rat (C, M, and T group) feces (≈50 mg) were mixed with 500 µL of extraction solution (acetonitrile: isopropyl alcohol: water, 3:3:2) and homogenized using a high‐flux tissue grinder (SCIENTZ, China). The samples were sonicated at 30 Hz for 20 s and ultrasonicated in an ice‐water bath for 5 min. Next, 500 µL of the extraction solution was added, and ultrasonication was performed in an ice‐water bath for 5 min. The supernatant was centrifuged (10000 × *g*, 5 min) and concentrated to dryness (8–10 h) in a vacuum concentrator. The samples were redissolved in 80 µL of 20 mg mL^−1^ methoxypyridine solution, vortexed for 30 s, and incubated at 60 °C for 60 min. Then, 100 µL of BSTFA‐TMCS (99:1) reagent was added, and the solution was incubated at 70 °C for 90 min. Finally, the solution was centrifuged (10 000 × *g*, 5 min), and the supernatant was collected for gas chromatography–time‐of‐flight mass spectrometry (GC‒TOF‒MS) analysis (Personalbio Biotechnology Co., Ltd., Shanghai, China).

Gas chromatography was performed using a DB‐5MS capillary column (Agilent J&W Scientific, Folsom, CA, USA) to separate derivatized substances at a constant helium flow rate of 1 mL min^−1^. One microliter of sample was injected through the autosampler at a split ratio of 1:10. The injection temperature was 280 °C, and the transfer line and ion source temperatures were 320 and 230 °C, respectively. The temperature conditions were as follows: the initial temperature was 50 °C for 0.5 min, followed by 15 °C min^−1^ ramping up to 320 °C and holding at 320 °C for 9 min. Mass spectrometry was performed under the full scanning method with a scanning rate of 10 spec s^−1^, electron energy of −70 V, and solvent delay of 3 min. The sequence of analysis of all test samples was random. After quality inspection, the samples were confirmed to meet the requirements, and then other experiments and bioinformatics analyses were performed.^[^ ^55^ ^]^

### Proteomic Analysis

Three biological replicates of EVs (EV1, EV2, and EV3) and GEVs (GEV1, GEV2, and GEV3) were obtained, and 4D‐label‐free quantitative proteomic analysis was performed by Personalbio Biotechnology Co., Ltd. (Shanghai, China). The proteins from the EVs and GEVs were extracted by the SDT (4% SDS, 100 mM Tris/HCl (pH 7.6), and 0.1 M DTT) cleavage method and quantified using a BCA protein assay kit. An appropriate amount of protein was extracted from each sample for trypsin digestion using the filter‐aided proteome preparation (FASP) method. Then, the peptides were desalted using a C18 cartridge, lyophilized, and reconstituted in 40 µL of 0.1% formic acid solution for peptide quantification (OD280). LC‒MS/MS analysis was performed using a timsTOF Pro mass spectrometer (Bruker, Germany) after chromatographic separation. Mass spectrometry data were analyzed using MaxQuant software (v.1.6.14) for library identification and quantitative analysis. Finally, bioinformatics analysis was performed, including protein cluster analysis (Cluster 3.0 and Java Treeview software), protein domain analysis (InterProScan software), GO functional annotation (NCBI BLAST + client and InterProScan software), KEGG pathway annotation, enrichment analysis, and protein‒protein interaction analysis (STRING and Cytoscape software). The mass deviation of all identified peptides was mainly distributed within 10 ppm, and for each set of label‐free data, qualitative peptide FDR ≤ 0.01 and protein FDR ≤ 0.01 were used as screening criteria, indicating that the results were accurate and reliable.^[^ ^17^ ^]^

### Statistical Analysis

Data were presented as the mean ± standard deviation (SD). GraphPad Prism software (version 7.0; San Diego, CA, USA) and SPSS software (version 17.0; SPSS Inc., Chicago, USA) were used for statistical analyses. Significance was determined using unpaired two‐tailed Student's *t* test for two groups, and one‐way analysis of variance (ANOVA) followed by Tukey's multiple comparison test was used for multiple groups. Data were considered significant at *p* < 0.05, with */#/▼ *p* < 0.05, **/##/▼▼ *p* < 0.01, ***/###/▼▼▼*p* < 0.001, and ****, *p* < 0.0001.^[^ ^56^ ^]^

## Conflict of Interest

The authors declare no conflict of interest.

## Author Contributions

J.L., W.X., C.‐H.Z. performed conceptualization; C.‐H.Z., Y.‐C.Z., H.‐L.X., W.‐M.Z., Z.‐Y.W., W.‐J.Z., X.‐G.P. performed methodology; C.‐H.Z., Y.‐C.Z., W.‐M.Z., J.X. performed visualization; C.‐H.Z., W.‐M.Z. wrote the original draft. All authors discussed the results and commented on the final version of the manuscript.

## Supporting information

Supporting Information

## Data Availability

The data that support the findings of this study are available from the corresponding author upon reasonable request.
