# Supplementary material for: Chlorogenic Acid Ameliorates Post‐Infectious Irritable Bowel Syndrome by Regulating Extracellular Vesicles of Gut Microbes
Source: Adv Sci (Weinh). 2023 Aug 24;10(28):2302798. doi: 10.1002/advs.202302798 (PMC10558682; doi:10.1002/advs.202302798)
Supplement: Supplementary file 1 — Supporting Information [file ADVS-10-2302798-s001.pdf]

ADVANCED SCIENCE
Open Access

Supporting Information

for *Adv. Sci.*, DOI 10.1002/advs.202302798

Chlorogenic Acid Ameliorates Post-Infectious Irritable Bowel Syndrome by Regulating Extracellular Vesicles of Gut Microbes

Cihua Zheng, Yuchun Zhong, Wenming Zhang, Zhuoya Wang, Haili Xiao, Wenjun Zhang, Jian Xie, Xiaogang Peng, Jun Luo and Wei Xu**

Supplementary materials

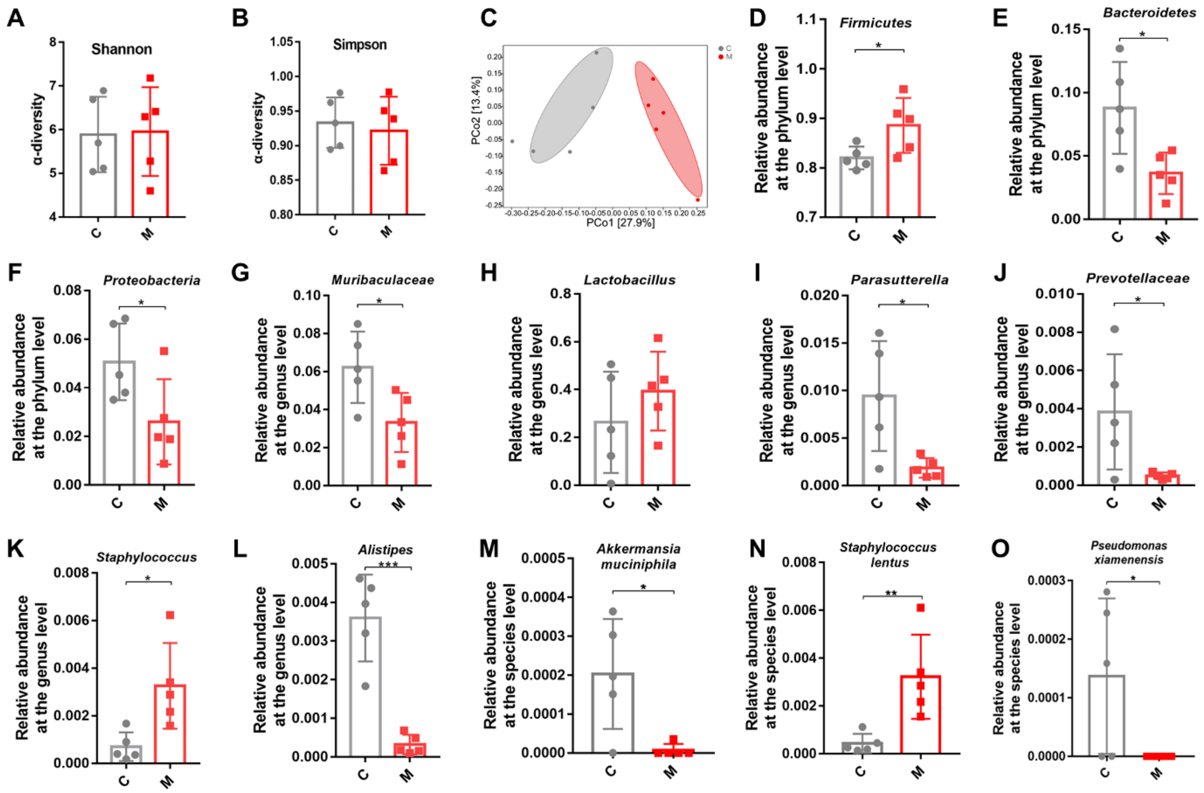

Figure S1. Composition of gut microbiota in rats with postirritable bowel syndrome. A) Shannon diversity. B) Simpson diversity. C) PCoA to estimate the β-diversity index (n=5). D) *Firmicutes*. E) *Bacteroidetes*. F) *Proteobacteria*. G) *Muribaculaceae*. H) *Lactobacillus*. I) *Parasutterella*. J) *Prevotellaceae*. K) *Staphylococcus*. L) *Alistipes*. M) *Akkermansia muciniphila*. N) *Staphylococcus lentus*. O) *Pseudomonas xiamenensis*. C, control group; M, PI-IBS rat group. Data are presented as the mean ± SD. * $P < 0.05$; ** $P < 0.01$; ***, $P < 0.001$.

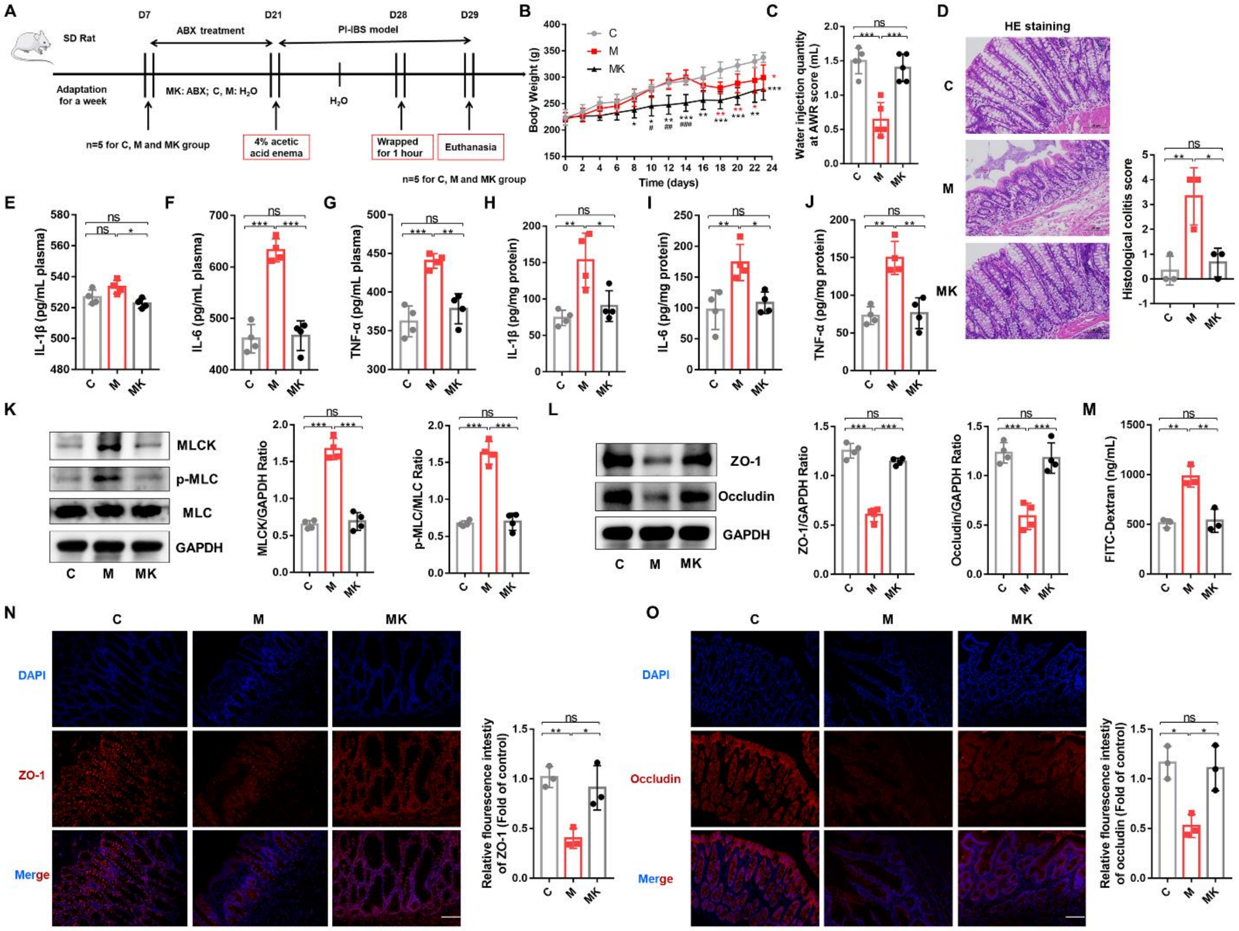

Figure S2. The gut microbiota was involved in the occurrence of PI-IBS. A) Schematic diagram of the effect of ABC on the PI-IBS model in rats (n=5). B) ABC reduced the weight of rats (n=5). C) Water injection quantity at an AWR score of 3 (n=5). D) H&E staining and pathological score of colon sections (n=3). E-J) Concentrations of the representative proinflammatory cytokines IL-1β, IL-6 and TNF-α in rat serum and colon tissue (n=4). K) Expression of key proteins of the MLCK/p-MLC signaling pathway in rat colon tissue (n=4). L) The expression of tight junction proteins (ZO-1, Occludin) in rat colon tissue (n=4). M) Measurement of gut permeability in rats (n=3). N, O) Immunofluorescence staining of tight junction protein (ZO-1, Occludin) expression in the colonic section (n=3). C, control group; M, PI-IBS rat group. Scale bar, 100 μm. Data are presented as the mean ± SD. ns, $P > 0.05$; * $P < 0.05$; ** $P < 0.01$; ***, $P < 0.001$. AA: Acetic acid; PI-IBS: Post-infectious irritable bowel syndrome; AWR: Abdominal withdrawal reflex; ABC: antibiotic cocktail.

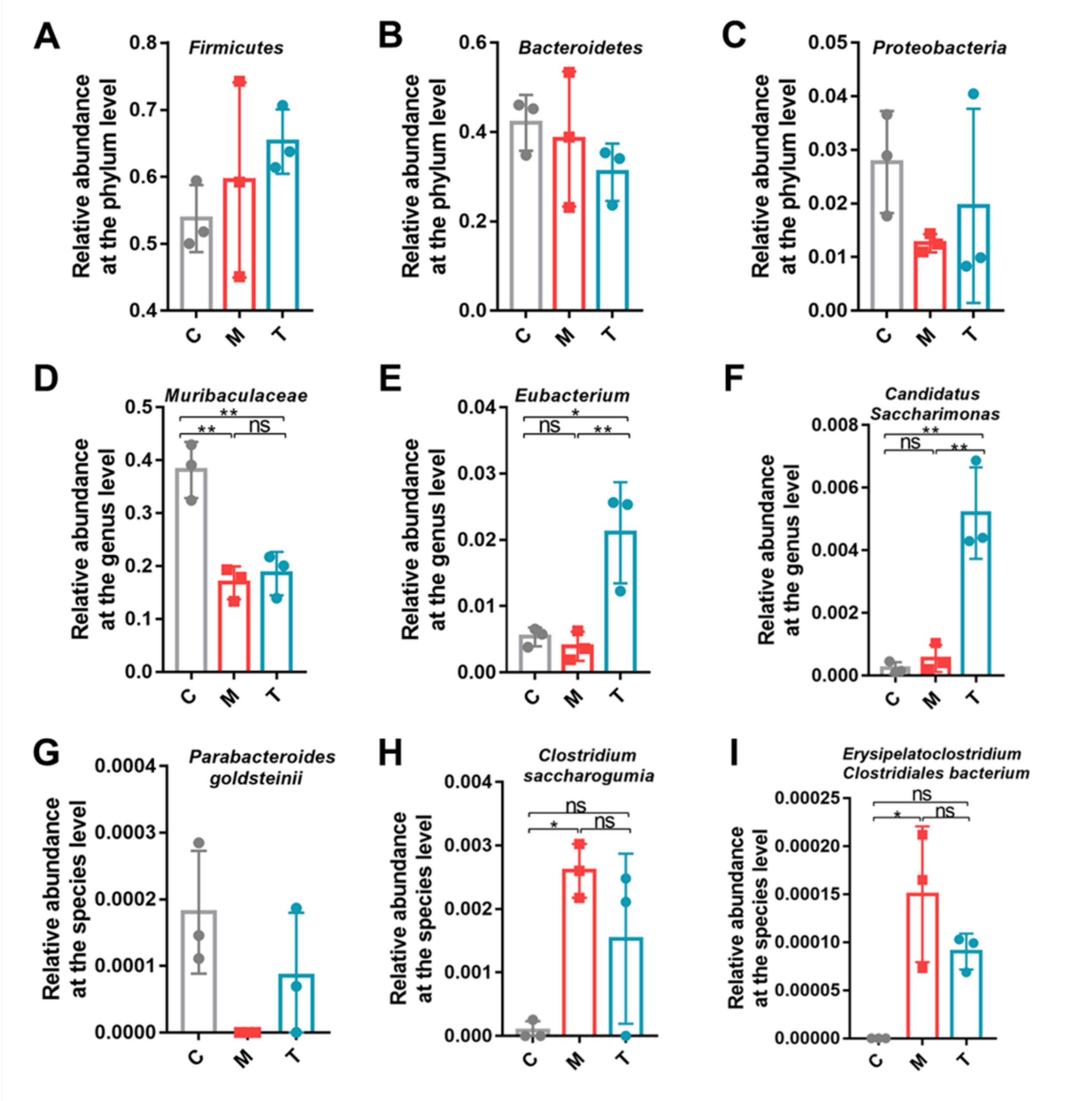

Figure S3. Effect of rectal administration of CGA on gut microbiota composition in rats. A) *Firmicutes*. B) *Bacteroidetes*. C) *Proteobacteria*. D) *Muribaculaceae*. E) *Eubacterium*. F) *Candidatus Saccharimonas*. G) *Parabacteroides goldsteinii*. H). *Clostridium saccharogumia*. I) *Erysipelatoclostridium Clostridiales bacterium*. (n=3). C, control group; M, PI-IBS rat group; T, PI-IBS rats rectally administered CGA group. Data are presented as the mean ± SD. ns, $P > 0.05$; * $P < 0.05$; ** $P < 0.01$. CGA: chlorogenic acid.

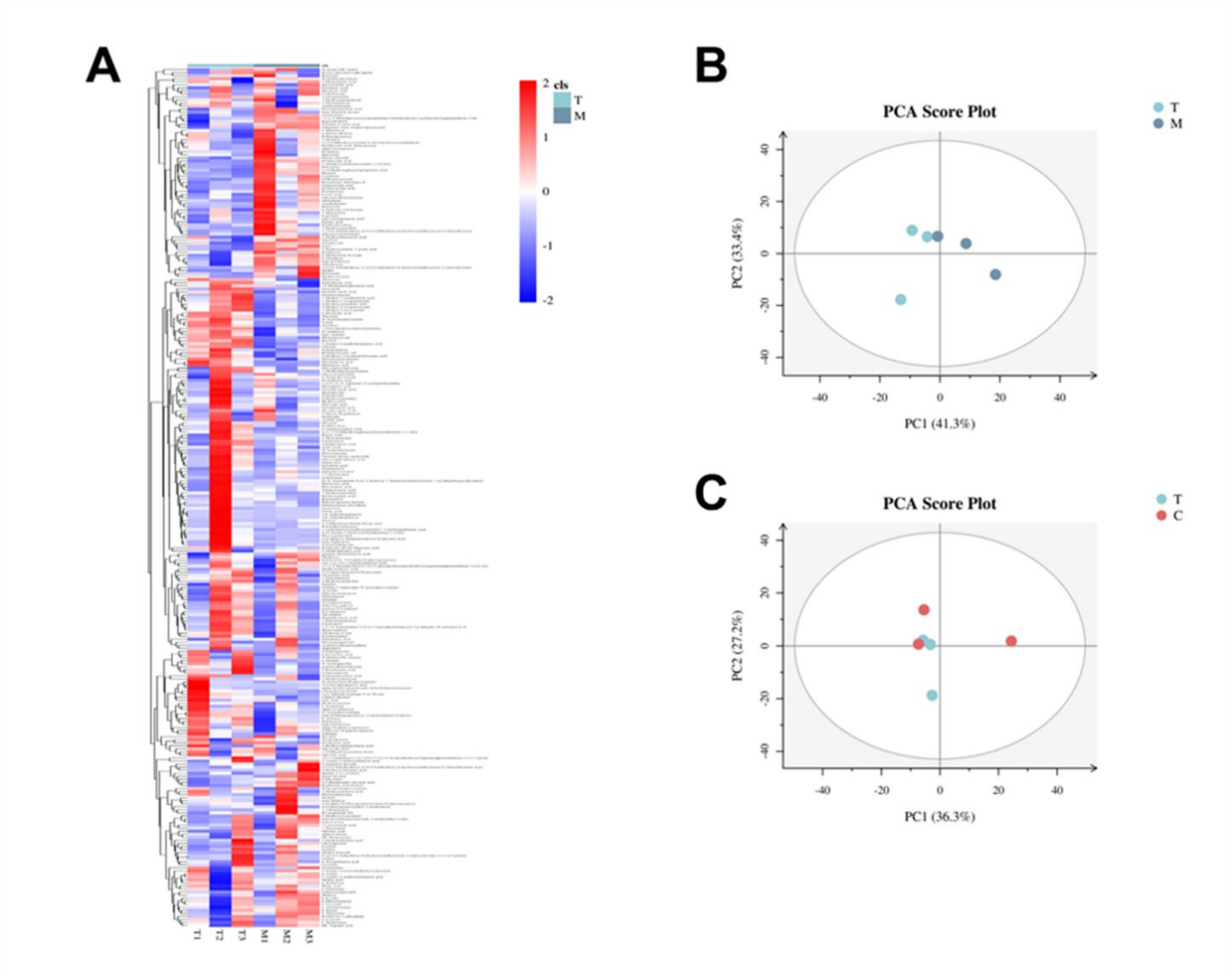

Figure S4. Effect of rectal administration of CGA on the composition of fecal metabolites in rats. A) The 282 metabolites analyzed were represented by a thermogram. B) Principal component analysis of metabolic data between the T and M groups. C) Principal component analysis of metabolic data between the T and C groups. The red dot represents the sample of the control group, the green dot represents the sample of the CGA treatment group, and the blue dot represents the sample of the model group. (n=3). C, control group; M, PI-IBS rat group; T, PI-IBS rats rectally administered CGA group. CGA: chlorogenic acid.

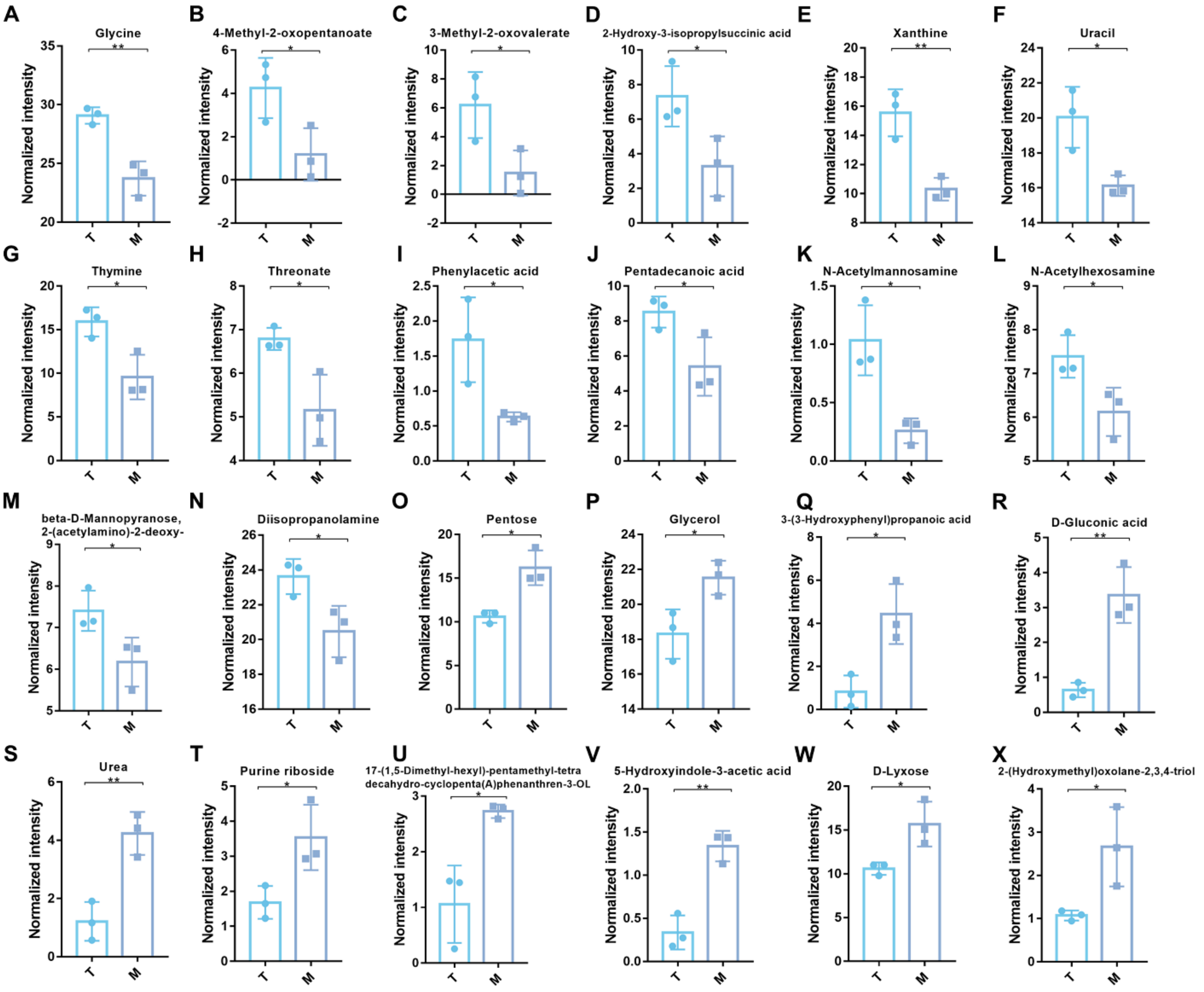

Figure S5. The levels of 24 metabolites changed significantly after CGA treatment of PI-IBS. Among these metabolites, the former 14 metabolites significantly increased and the latter 10 metabolites obviously decreased after treatment. The ordinate represents the peak area, the green bar represents the CGA treatment group sample, and the blue bar represents the PI-IBS group sample. (n=3). M, PI-IBS rat group; T, PI-IBS rats rectally administered CGA group. Data are presented as the mean ± SD. * $P < 0.05$; ** $P < 0.01$. CGA: chlorogenic acid.

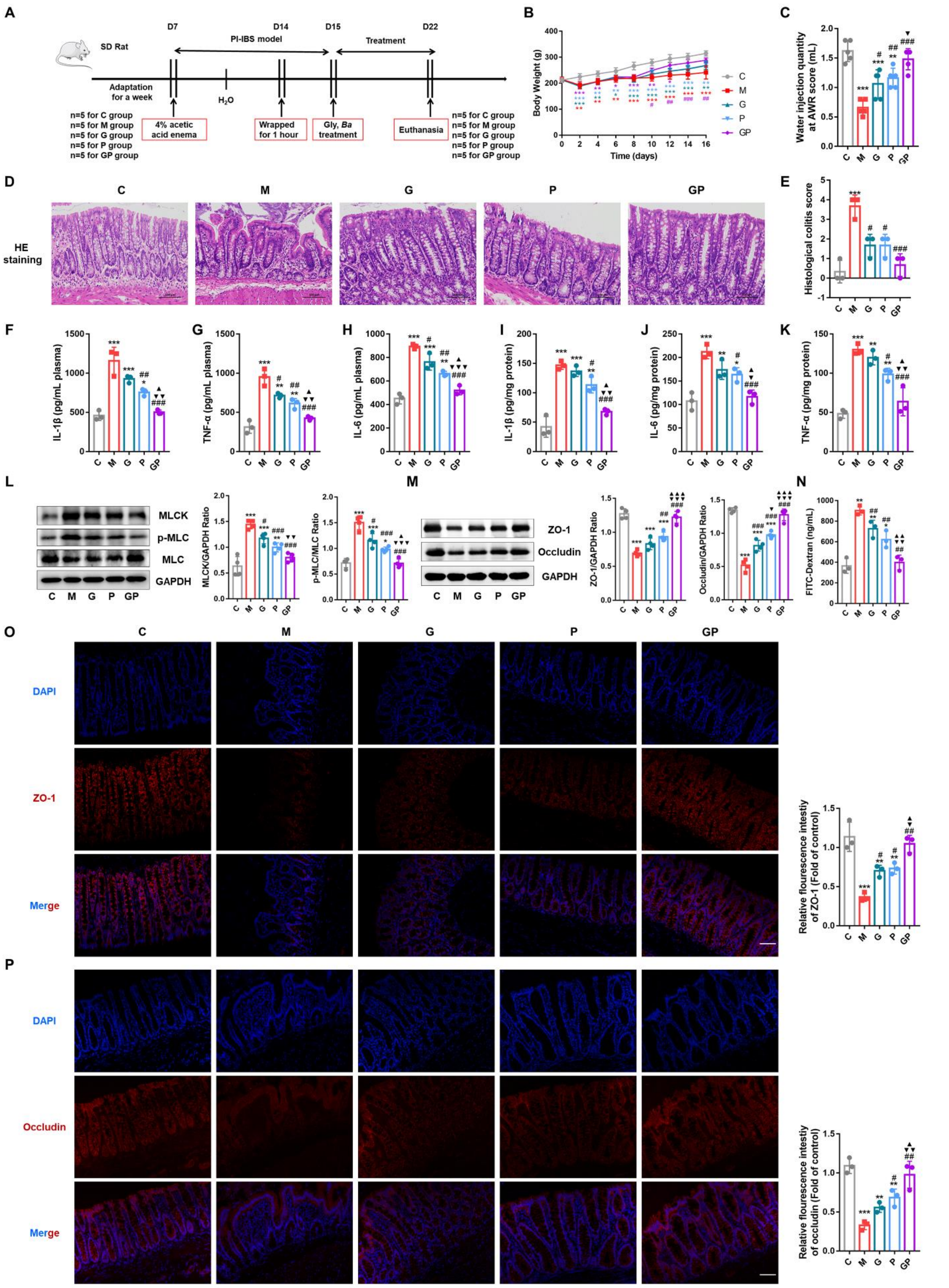

Figure S6. Concomitant use of *B. acidifaciens* and glycine relieved symptoms of PI-IBS in rats A) Schematic diagram of PI-IBS rats administered *B. acidifaciens* and glycine. B) Changes in body weight (n=5). C) Water injection quantity at an AWR score of 3 (n=5). D, E) H&E staining

and pathological score of colon sections (n=3). F-K) Concentrations of the representative proinflammatory cytokines IL-1β, IL-6 and TNF-α in rat serum and colon tissue (n=3). L) Expression of key proteins of the MLCK/p-MLC signaling pathway in rat colon tissue (n=4). (M) The expression of tight junction proteins (ZO-1, Occludin) in rat colon tissue (n=3-4). N) Measurement of gut permeability in rats (n=3). O, P) Immunofluorescence staining of tight junction protein (ZO-1, Occludin) expression in the colonic section (n=3). C, control group; M, PI-IBS rat group; G, free drinking 0.5% glycine water group; P, 1×10^9 CFU *B. acidifaciens* rectal administration group; GP, glycine and *B. acidifaciens* combination treatment group. Scale bar, 100 μm. Data are presented as the mean ± SD. * $P < 0.05$ versus C group, # $P < 0.05$ versus M group, ▼$P < 0.05$ versus G group, ▲ $P < 0.05$ versus P group. ns, $P > 0.05$; */#/▼/▲ $P < 0.05$; **/##/▼▼/▲▲ $P < 0.01$; ***/###/▼▼▼/▲▲▲$P < 0.001$. The expression of tight junction proteins (ZO-1, Occludin) in rat colon tissue (n=4).

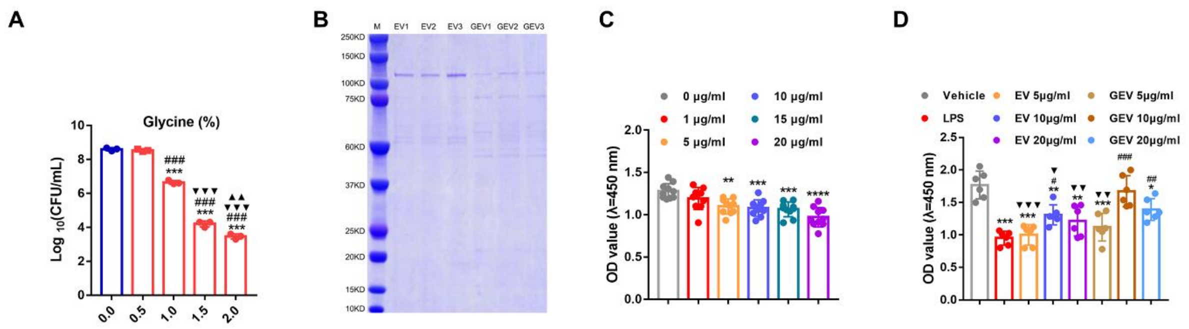

Figure S7: A) Viability of *B. acidifaciens* treated with glycine at various concentrations (0%, 0.5%, 1%, 1.5%, and 2%). B) EV and GEV protein profiles. C, D) EV treatment reversed LPS-induced cytotoxicity of Caco-2 cells as measured by Cell Counting Kit-8 assay. (n=10; n=6). Data are presented as the mean ± SD. * $P < 0.05$ versus 0% or vehicle group, # $P < 0.05$ versus 0.5% or LPS group, ▼$P < 0.05$ versus 1.0% or GEV 10 μg/mL group, ▲ $P < 0.05$ versus 1.5%. ns, $P > 0.05$; */#/▼ $P < 0.05$; **/##/▼▼ $P < 0.01$; ***/###/▼▼▼$P < 0.001$; ****, $P < 0.0001$. EV: without induced *B. acidifaciens* extracellular vesicles; GEV: glycine-induced *B. acidifaciens* extracellular vesicles.

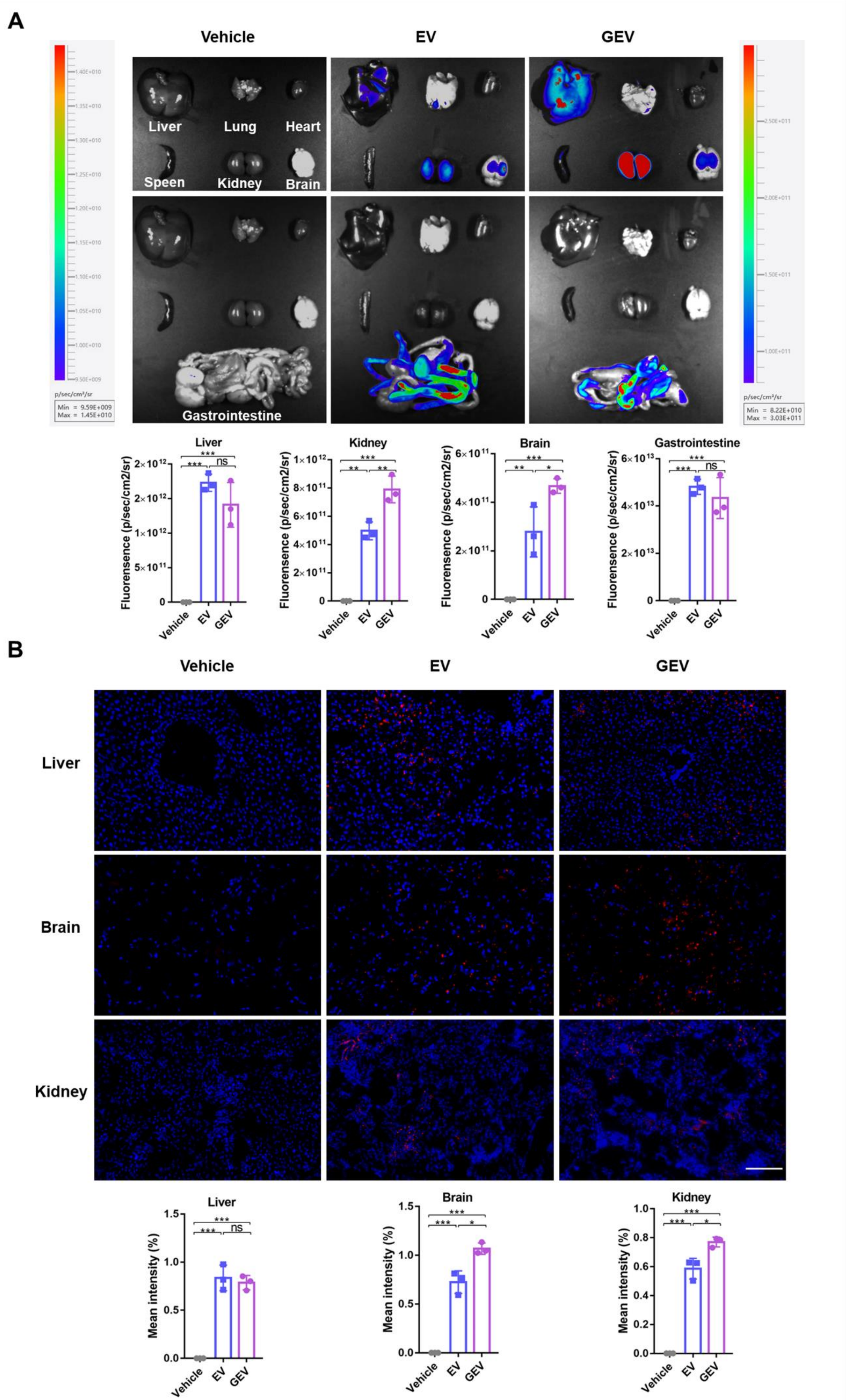
A
Vehicle
EV
GEV
Liver
Lung
Heart
Speen
Kidney
Brain
Gastrointestine
p/sec/cm²/sr
Min = 9.59E+009
Max = 1.45E+010
Min = 8.22E+010
Max = 3.03E+011
Liver
Kidney
Brain
Gastrointestine
Fluorensence (p/sec/cm2/sr)
Vehicle
EV
GEV
ns
B
Vehicle
EV
GEV
Liver
Brain
Kidney
Liver
Brain
Kidney
Mean intensity (%)

Figure S8. Biodistribution of EVs in rats. A) EV *in vivo* fluorescence imaging of rat lung, kidney, heart, spleen, brain, liver, and gastrointestinal tract treated with vehicle or Dil-labeled EVs (EV and GEV) for 3 hours and quantification of the fluorescent signals (n=3). B) Fluorescence microscopy analysis of kidney, brain and liver sections from rats treated with PKH26-labeled EVs for 3 hours by rectal administration and quantification of the fluorescent signals (n=3). Scale bar, 100 μm. Data are presented as the mean ± SD. ns, $P > 0.05$; * $P < 0.05$; ** $P < 0.01$; ***, $P < 0.001$. EV: without induced *B. acidifaciens* extracellular vesicles; GEV: glycine-induced *B. acidifaciens* extracellular vesicles.

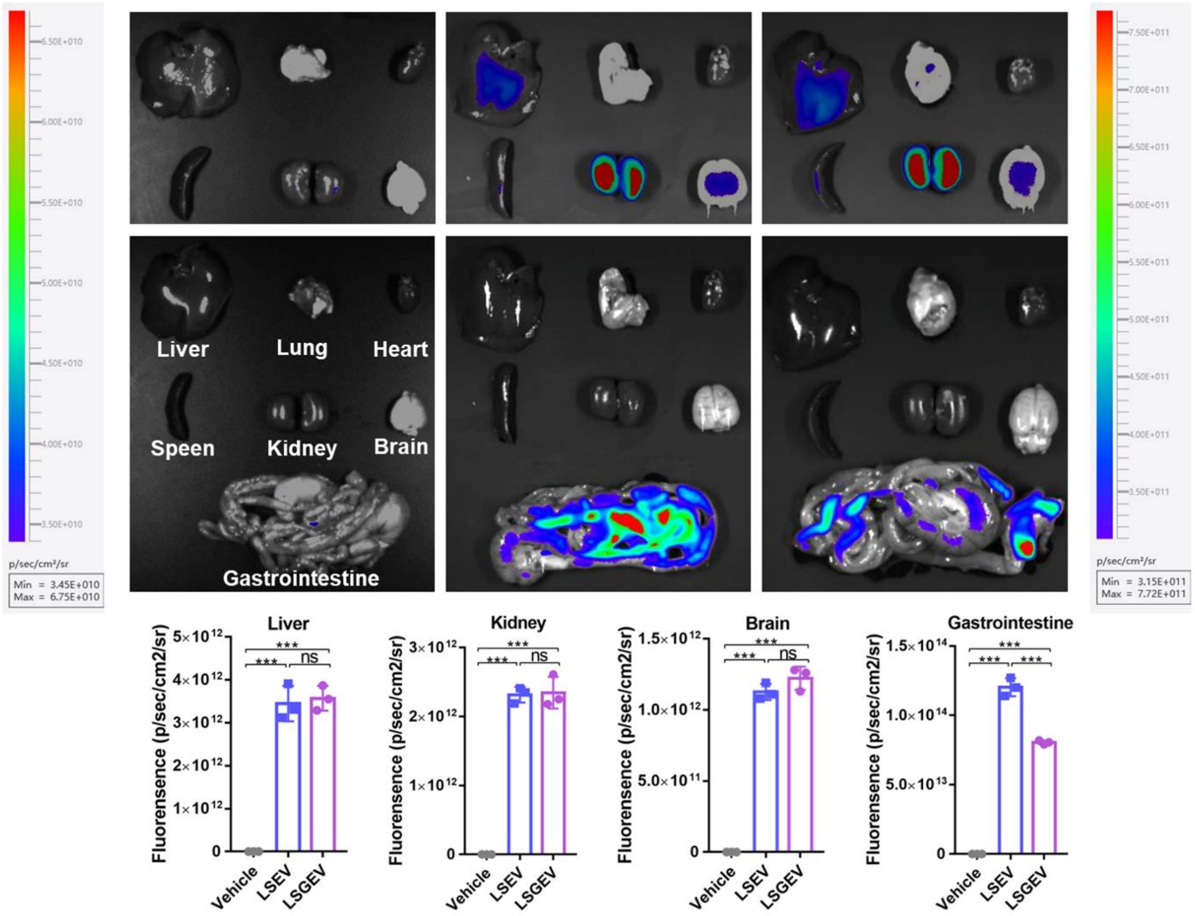

Figure S9. Biodistribution of *Lactobacillus salivarius* EVs in rats. *L. salivarius* EV *in vivo* fluorescence imaging of rat lung, kidney, heart, spleen, brain, liver, and gastrointestinal tract treated with vehicle or Dil-labeled *L. salivarius* EVs (LSEV and LSGEV) for 3 hours and quantification of the fluorescent signals (n=3). Data are presented as the mean ± SD. ns, $P > 0.05$; ***, $P < 0.001$. LSEV: without induced *L. salivarius* extracellular vesicles; LSGEV: 0.5% glycine-induced *L. salivarius* extracellular vesicles.

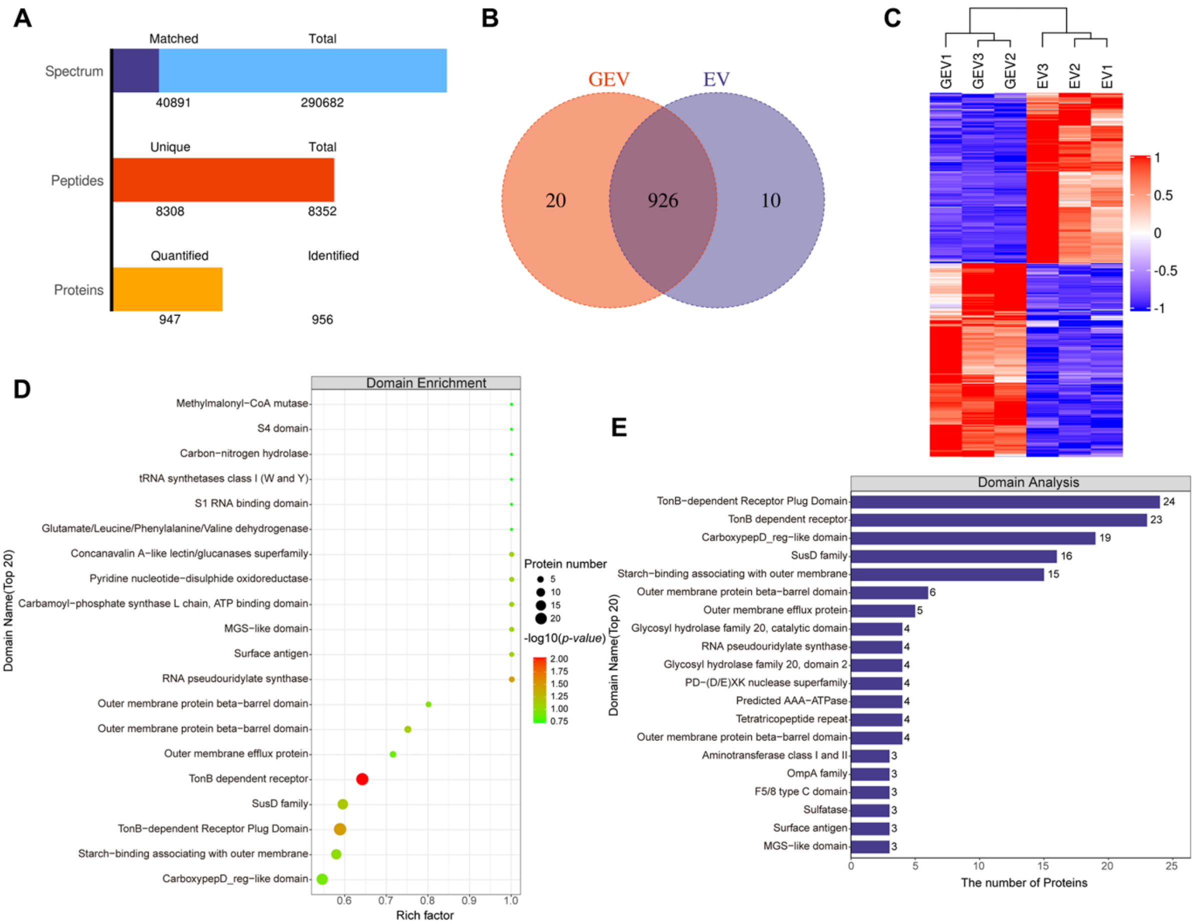

Figure S10. Proteomic analysis of GEV and EV. A) Summary of tandem mass spectrometry (MS/MS) database search results for GEV and EV. B) Scalar-Venn representation. C) Protein cluster analysis of differential expression between GEV and EV. Each column represents a group of samples, and each row represents a protein. The expression of sexually different proteins in different samples was standardized by the Z Score method and displayed in a heatmap in different colors, in which red represents proteins with a significant increase, blue represents proteins with a significant decrease, and gray represents proteins with no quantitative information. D) Plots of enrichment analysis of GEV and EV domains. The abscissa in the figure represents the enrichment factor (rich factor ≤ 1). The vertical axis represents the statistical results of differential proteins under each domain classification. Bubble colors indicate the importance of rich domain classification. *P* values were calculated according to Fisher's exact test, and the color gradient indicates the magnitude of the *P* value. The closer the color is to red, the smaller the *P* value, and the higher the significance level of enrichment under the corresponding domain classification. E) Domain analysis of differentially expressed proteins in GEV and EV. The number of the top 20 proteins in the domain is displayed as a histogram. (n=3). EVs: extracellular vesicles; EV: without induced *B. acidifaciens* extracellular vesicles; GEV: glycine-induced *B. acidifaciens* extracellular vesicles.

Supplementary Table 1 Top 24 metabolites in fecal samples from normal control (C group) or PI-IBS rats (M group).

Metabolites	VIP-value	*P*-value
5-Hydroxyindole-3-acetic acid	1.185603746	0.002806557
D-Gluconic acid	1.940911626	0.004610574
Glycine	2.723823414	0.004629623
Urea	2.0361113	0.006242666
Xanthine	2.674386508	0.007178098
Pentose	2.742432452	0.010139166
N-Acetylmannosamine	1.014587074	0.013479445
17-(1,5-Dimethyl-hexyl)-pentamethyl-tetradecahydro-cyclopenta(A)phenanthren-3-OL	1.484142339	0.014893969
3-(3-Hydroxyphenyl) propanoic acid	2.171669328	0.016777492
Uracil	2.246513727	0.021080723
Thymine	2.847779096	0.022984309
Threonate	1.434369241	0.029294302
D-Lyxose	2.528131224	0.02940553
Glycerol	2.007253247	0.031131913
Phenylacetic acid	1.167782868	0.035187434
Purine riboside	1.51195004	0.036964422
Diisopropanolamine	1.97001242	0.037560594
N-Acetylhexosamine	1.244244937	0.040344592
2-(Hydroxymethyl) oxolane-2,3,4-triol	1.392309466	0.040796833
3-Methyl-2-oxovalerate	2.388649173	0.042471219
4-Methyl-2-oxopentanoate	1.922228687	0.045444627
Pentadecanoic acid	1.934954653	0.046273047
2-Hydroxy-3-isopropylsuccinic acid	2.207127272	0.046447917
beta-D-Mannopyranose, 2-(acetylamino)-2-deoxy-	1.213310773	0.048821614

P-values were determined using a two-sided, Fisher's exact test with Benjamini-Hochberg multiple-testing correction

Supplementary Table 2. The main metabolic pathways analysis.

Pathway	Raw p	-log(p)	Impact
Pyrimidine metabolism	0.010854	4.5232	0.076506
Valine, leucine and isoleucine biosynthesis	0.011585	4.458	0.12903
Purine metabolism	0.029845	3.5118	0.039711
Valine, leucine and isoleucine degradation	0.03627	3.3168	0.11858
Pentose and glucuronate interconversions	0.061028	2.7964	0.017241
Phenylalanine metabolism	0.068933	2.6746	0.035714
ABC transporters	0.075474	2.584	0.021739
Synaptic vesicle cycle	0.083634	2.4813	0.083333
Regulation of lipolysis in adipocytes	0.096904	2.334	0.14286
Retrograde endocannabinoid signaling	0.12928	2.0458	0.052632
Caffeine metabolism	0.14817	1.9094	0.029412
Arginine biosynthesis	0.15438	1.8684	0.018868
Thermogenesis	0.15438	1.8684	0.10256
Biotin metabolism	0.18477	1.6886	0.015625
Mineral absorption	0.19073	1.6569	0.028571
Pantothenate and CoA biosynthesis	0.19664	1.6264	0.026786
Thiamine metabolism	0.20251	1.597	0.022222
beta-Alanine metabolism	0.20833	1.5686	0.025862
Pentose phosphate pathway	0.22557	1.4891	0.041026
Central carbon metabolism in cancer	0.23687	1.4403	0.018868
Glutathione metabolism	0.24245	1.4169	0.048951
Glycerolipid metabolism	0.24245	1.4169	0.12987
Serotonergic synapse	0.26441	1.3302	0.016129
Galactose metabolism	0.28576	1.2526	0.015873
Protein digestion and absorption	0.29101	1.2344	0.021277
Primary bile acid biosynthesis	0.29101	1.2344	0.04878
Lysine degradation	0.30652	1.1825	0.006493
Ascorbate and aldarate metabolism	0.30652	1.1825	0.006493
Glycine, serine and threonine metabolism	0.30652	1.1825	0.096273
Aminoacyl-tRNA biosynthesis	0.31668	1.1499	0.017857
Neuroactive ligand-receptor interaction	0.31668	1.1499	0.019231
Phosphonate and phosphinate metabolism	0.33657	1.089	0.014706
Glyoxylate and dicarboxylate metabolism	0.36537	1.0068	0.10769
Arginine and proline metabolism	0.43643	0.82912	0.068627
Tryptophan metabolism	0.45703	0.783	0.042296
Amino sugar and nucleotide sugar metabolism	0.54965	0.59847	0.065517
Porphyrin and chlorophyll metabolism	0.65162	0.42829	0.019608
